# Fate of MHCII in salmonids following 4WGD

**DOI:** 10.1007/s00251-020-01190-6

**Published:** 2020-11-23

**Authors:** Unni Grimholt, Morten Lukacs

**Affiliations:** Norwegian Veterinary Institute, P.O. Box 8146 Dep, 0033 Oslo, Norway

**Keywords:** MHC class II, Salmonids, Northern pike, Evolution

## Abstract

Major histocompatibility complex (MHC) genes are key players in the adaptive immunity providing a defense against invading pathogens. Although the basic structures are similar when comparing mammalian and teleost MHC class II (MHCII) molecules, there are also clear-cut differences. Based on structural requirements, the teleosts non-classical MHCII molecules do not comply with a function similar to the human HLA-DM and HLA-DO, i.e., assisting in peptide loading and editing of classical MHCII molecules. We have previously studied the evolution of teleost class II genes identifying various lineages and tracing their phylogenetic occurrence back to ancient ray-finned fishes. We found no syntenic MHCII regions shared between cyprinids, salmonids, and neoteleosts, suggesting regional instabilities. Salmonids have experienced a unique whole genome duplication 94 million years ago, providing them with the opportunity to experiment with gene duplicates. Many salmonid genomes have recently become available, and here we set out to investigate how MHCII has evolved in salmonids using Northern pike as a diploid sister phyla, that split from the salmonid lineage prior to the fourth whole genome duplication (4WGD) event. We identified 120 MHCII genes in pike and salmonids, ranging from 11 to 20 genes per species analyzed where DB-group genes had the most expansions. Comparing the MHC of Northern pike with that of Atlantic salmon and other salmonids species provides a tale of gene loss, translocations, and genome rearrangements.

## Introduction

In mammals, the core major histocompatibility complex (MHC) represents one gene dense genomic region of approximately 4 mega bases that contains MHC class I (MHCI) and MHC class II (MHCII) genes in addition to many other genes with or without immune function (Horton et al. 2004). The MHC genes themselves encode two different classes of molecules, MHC class I (MHCI) and MHC class II (MHCII), with many different genes within each class. Each MHC class is divided into either classical or non-classical genes where classical genes are highly polymorphic peptide-binders and non-classical genes deviate from this rule.

Classical MHCI genes are expressed on most cells, encode molecules that bind and present self, and endogenously derived peptides to CD8 positive T cells, thus initiating a cellular immune response towards invading pathogens (Klein 1986). Classical MHCII genes encode molecules that in general bind and present exogenously derived peptides to CD4 positive T cells, thus initiating a humoral immune response towards the invading pathogens (Klein 1986). A classical MHCII gene is further defined by expression primarily in professional antigen presenting cells such as B-cells, macrophages, and dendritic cells, with subsequent restricted tissue expression patterns. Human MHCII genes encoding classical molecules are the HLA-DR, HLA-DQ, and HLA-DP alpha and beta genes Structurally, MHCII molecules are composed of an alpha as well as a beta chain each with two extracellular domains, a transmembrane domain, and a cytoplasmic tail. Polymorphism of classical genes primarily resides in the alpha 1 and beta 1 domains, while the alpha 2 and beta 2 domains contribute with molecular structure and CD4 association. Human non-classical MHCII genes, i.e., HLA-DM and HLA-DO, are not polymorphic and do not bind peptides. Instead, they assist in the peptide loading of their classical counterpart.

In humans, MHCII is associated with many autoimmune diseases such as celiac disease, rheumatoid arthritis, and narcolepsy (Thorsby and Lie 2005). Associations with infectious diseases are more debated although MHCII seems to have an effect for instance on disease progression for hepatitis (Blackwell 2009). It is speculated that the close linkage between MHCI and MHCII genes and multiple classical MHC genes reduces the selection pressure on individual MHCI and MHCII alleles, providing haplotypes that perform well against most infectious pathogens (Satta et al. 1994).

In mammals, constitutive MHCII expression is found in professional antigen presenting cells controlled by a canonical MHCII promoter consisting of a S-X-Y sequence module that is bound by enhanceosome transcription factors consisting of RFX5, RFXAP, RFXANK, CREB/ATF1, NF-Ys, and the MHCII transactivator CIITA (Meissner et al. 2012; Sachini and Papamatheakis 2017). Following transcription, the translated alpha and beta chain proteins assemble in the endoplasmatic reticulum, stabilized by the chaperone invariant chain (Ii) (reviewed in Neefjes et al. 2011). This MHCII/Ii complex is then transported to a late endosomal compartment termed the MHCII compartment (MIIC). Here, the peptidases cathepsin S (CTSS) and L (CTSL) contribute to Ii digestion into a class II associated Ii peptide denoted CLIP and also contribute to the peptide repertoire available for MHCII (Hsieh et al. 2002). In the MIIC, the non-classical MHCII molecules HLA-DM assists in exchange of the CLIP fragment for other peptides originating from the endosomal pathway while HLA-DO is a regulator of HLA-DM function (Alvaro-Benito and Freund 2020).

MHCII gene sequences have been identified in a vast number of teleosts where the molecular structure is similar to the mammalian counterpart (Conejeros et al. 2008; Dixon et al. 1996; Godwin et al. 1997; Grimholt et al. 2000; Harstad et al. 2008; McConnell et al. 1998; Ono et al. 1992, 1993; Sato et al. 2006, 2012; Sultmann et al. 1993, 1994; Van Erp et al. 1996; Walker and McConnell 1994). In a few species, such as Atlantic salmon and rainbow trout, classical and non-classical molecules are defined where classical MHCII genes are highly polymorphic and non-classical genes have low polymorphism and more restricted expression patterns (Grimholt et al. 2003; Harstad et al. 2008; Landry and Bernatchez 2001; Shum et al. 2001).

There are a few teleost species that do not have functional MHCII molecules, such as gadoids, pipefishes, and potentially some anglerfish, that have lost a functional MHCII system (Dubin et al. 2019; Haase et al. 2013; Small et al. 2016; Star et al. 2011). These species are all neoteleosts so the loss of MHCII seems to have occurred independently in several species. How these species mount a humoral response for instance against bacterial pathogens remains to be established, but it has been suggested that they expanded MHCI where some MHCI molecules have replaced the MHCII function (Malmstrom et al. 2013; Miller et al. 2002).

A major difference between mammalian and teleost MHC is that in teleosts the classical MHCI and MHCII genes have separated with class I being linked to genes involved in peptide generation and transport, while MHC class II genes reside elsewhere (Bingulac-Popovic et al. 1997; Grimholt 2016). The MHC class II most likely translocated out of the major MHC region prior to the split between teleosts and older ray-finned fishes. MHCI and MHCII genes are linked in Spotted gar, a non-teleost species that has not experienced the third WGD (Dijkstra et al. 2013). This lack of linkage, in addition to mostly single copy classical genes, has enabled stronger selection pressures to act upon individual MHCI and MHCII genes in teleosts.

Teleost fish MHCII molecules are defined through phylogenetic analyses into three lineages denoted DA, DB, and DE (Bannai and Nonaka 2013; Dijkstra et al. 2013). In salmonids, DE lineage genes reside alongside typical MHC region genes and their ancient nature is supported by the fact that they can be traced as far back as paddlefish and sturgeon. At least in Atlantic salmon, the DE lineage seems to be deteriorating with low expression levels and pseudogenes. DA and DB lineage genes originated from DE lineage genes in a primordial teleost potentially as a result of the teleost specific third whole genome duplication event (3WGD), as these lineages are not apparent in primitive bony fishes. DA and DB lineage genes have since diversified in some teleosts where the DB lineage or also called DB group is not a true lineage but contains several subgroups of sequences.

Classical MHCII genes in addition to some non-classical belong to the DA lineage. Although potentially not a true lineage, the DB lineage only consists of non-classical genes with low polymorphism and tissue-specific expressed pattern. None of the non-classical teleost MHCII molecules have the structural requirements to function as equivalents to the human non-classical HLA-DM and HLA-DO molecules, so their exact function remains undefined (Dijkstra et al. 2013).

Although three lineages have been defined for MHC class II, not all lineages are present in all species and the gene numbers vary dramatically. All analyzed teleost with MHCII have at least one functional DA lineage molecule, while the number of DB group genes range from zero to 16 (Dijkstra et al. 2013).

The assumed translocation of MHCII genes from the primordial teleost MHCI/II region seems to be representative for how MHCII genes have evolved in teleosts. We previously found no syntenies between MHCII regions in cyprinids, salmonids, and neoteleosts suggesting the MHCII genes have translocated to different regions in different species (Dijkstra et al. 2013). On the other hand, neoteleost such as tilapia, stickleback, and medaka displayed some regional MHCII syntenies, suggesting MHCII is more stable in neoteleosts than in cyprinids and salmonids. Gadoids could be an ultimate representative for such translocation processes where the entire MHCII machinery has been lost following translocation (Star et al. 2011).

Salmonids experienced a whole genome duplication 94 million years ago where many of the duplicated genes are retained as functional copies (Lien et al. 2016; Macqueen and Johnston 2014). And at least in Atlantic salmon, genes originating from the 4WGD have often taken on new functions rather than sub-functions of their duplicates (Lien et al. 2016). As Northern pike represents a sister phylum to salmonids, that split from the salmonid lineage prior to the fourth whole genome duplication (4WGD) event (Rogers et al. 2005), pike enables studies of how the 4WGD affected evolution of genes and gene duplicates in salmonids. Here, we make use of the available genomes of Northern pike and seven salmonids to study how the 4WGD affected the evolution of MHCII.

## Materials and methods

### Materials

Genomes used in this study originate from NCBI (National Center for Biotechnology, Bethesda, Maryland, USA, https://www.ncbi.nlm.nih.gov/genome/) are as follows: *Salvelinus alpinus*/*malma* GCA_002910315.2 (charr (Christensen et al. 2018b)), *Salmo trutta* GCA_901001165.1 (brown trout), *Oncorhynchus nerka* GCA_006149115.1 (sockeye salmon), *Oncorhynchus tshawytscha* GCA_002872995.1 (chinook salmon (Christensen et al. 2018a)), *Oncorhynchus kisutch* GCA_002021735.2 (coho salmon), *Oncorhynchus mykiss* Swanson river specimen GCA_002163495.1 (rainbow trout (Berthelot et al. 2014) and Arlee specimen genome GCA_013265735.1), and *Salmo salar* GCA_000233375.4 (Atlantic salmon (Lien et al. 2016)). For *Esox Lucius* the main genome used was GCA_004634155.1 (Northern pike (Rondeau et al. 2014)), while four additional genome assemblies available in the NCBI Genomes database were also used (GCA_007844535.1, GCA_000721915.3, GCA_011004835.1, GCA_011004845.1). NCBI genomes from Coregonus (GCA_902810595.1), *Hucho hucho* (GCA_003317085.1), and *Thymallus thymallus* (GCA_004348285.1) were not included, as they were not yet annotated.

### Data mining

Classical Atlantic salmon and rainbow trout MHCII alleles are gathered in the immune-polymorphism database for MHC (IPD-MHC database (https://www.ebi.ac.uk/ipd/mhc/)). Genome searches were performed using previously identified Atlantic salmon MHC amino acid gene sequences (Dijkstra et al. 2013; Harstad et al. 2008) and tblastn against NCBI resources. Genomic regions identified through these searches were screened for annotated genes. Some additional unannotated MHCII genes were identified using blast searches. Expressed match was identified through tblastn search using the entire coding sequence against EST or TSA/ SRA resources in NCBI.

### Sequence alignments and phylogenies

Mature extracellular amino acid domain sequences used in phylogenies were extracted using Jalview (Waterhouse et al. 2009). Amino acid sequences were aligned using ClustalX (Larkin et al. 2007) with manual correction. The evolutionary history of selected amino acid sequences was inferred by Neighbor-Joining method (Saitou and Nei 1987). Additional trees were also made by using the Maximum Likelihood method based on the JTT matrix-based model (Jones et al. 1992) or Whelan And Goldman model (Whelan and Goldman 2001). See Additional File 4 for further details. Evolutionary analyses were conducted in MEGA X (Kumar et al. 2018).

### Nomenclature

The nomenclature for MHC genes follows the suggestion from Klein et al. (1990). The first letter corresponds to D (duo) for class II, the second letter designates the locus (starting from A) and the third letter specifies A or B for class II alpha or II beta, respectively. Salmonid MHCII nomenclature used here originates from Harstad et al. (2008) where the DA lineage is represented by DAA and DAB genes, the DB group by DBB/DBA and DCB/DCA and DDA/DDB genes, and the DE lineage by DEA/DEB and DFA/DFB genes. Species abbreviations are included prior to the gene name as follows: Eslu for *Esox Lucius* (Northern pike), Sasa for *Salmo salar* (Atlantic salmon), Onmy for *Oncorhynchus mykiss* (rainbow trout), Onts for *Oncorhynchus tshawytscha* (chinook salmon), Onne for *Oncorhynchus nerka* (sockeye salmon), and Onki for *Oncorhynchus kisutch* (coho salmon). There seems to be some confusion as to the origin of the NCBI charr genome, now annotated as *Salvelinus* in NCBI, which may potentially be *Salvelinus malma malma* and not *Salvelinus alpinus* as presented in the original article (Christensen et al. 2018b; Shedko 2019). Here we chose to use Saal for *Salvelinus alpinus* although it may be Sama for *Salvelinus malma*. Deduced MHCII amino acid sequences from each genome are gathered in Additional file 1.

## Results and discussion

### Genomes analyzed and orthology between species

NCBI genomes from the salmonids Atlantic salmon, brown trout, rainbow trout, sockeye salmon, coho salmon, chinook salmon and Arctic charr, and Northern pike have been used for this study (see “Material and Methods” for details).

**Fig. 1:**
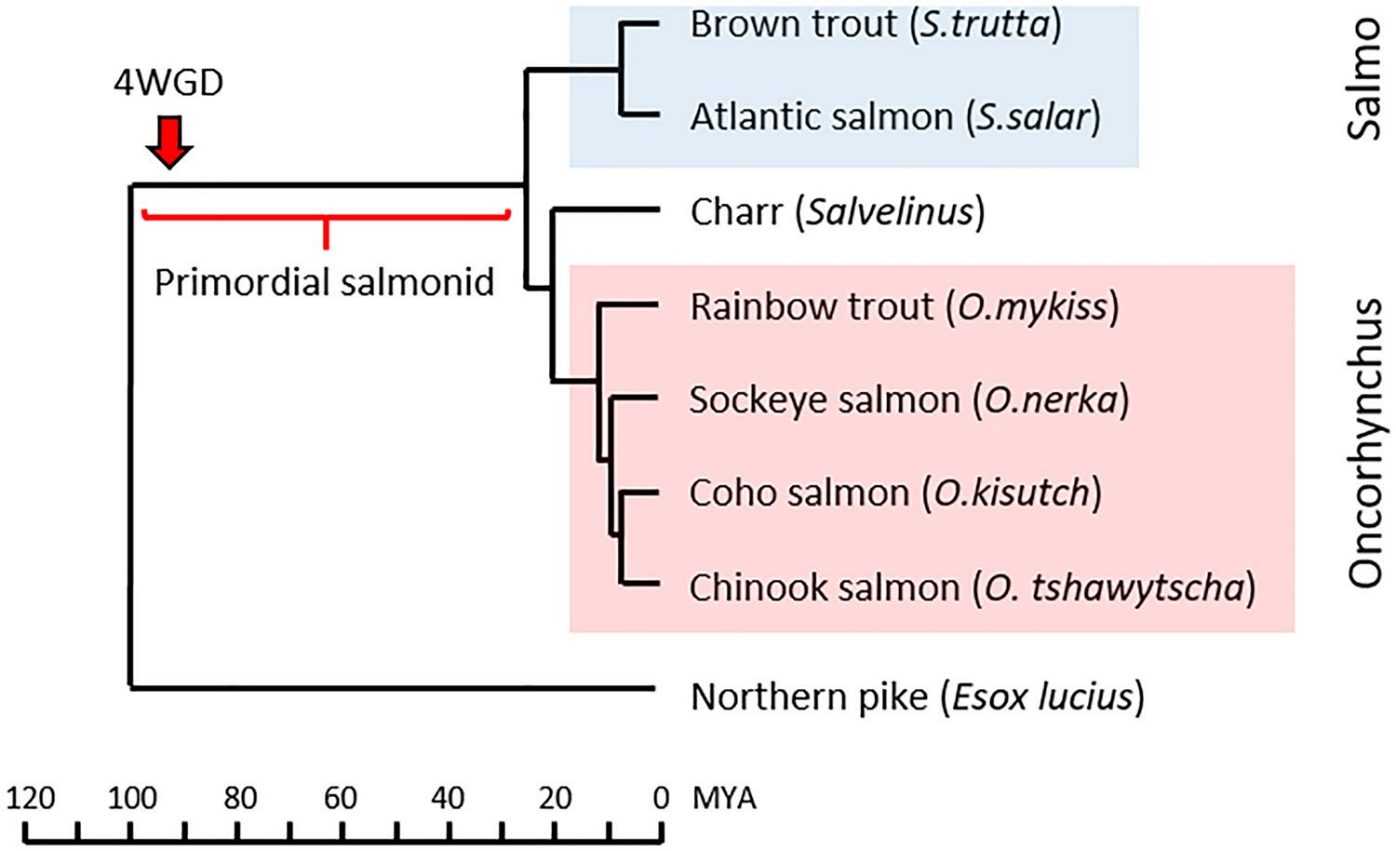
Phylogenetic relationships of Northern pike and selected salmonids. Schematic phylogenetic relationship between included species drawn with relative timing based on references data from (Crete-Lafreniere et al. 2012; Macqueen et al. 2017). Salmo and Oncorhynchus species are shown using a blue and red box respectively. The unique salmonid whole genome duplication event that occurred approximately 94 million years ago (MYA) (Macqueen and Johnston 2014) is shown using a red arrow

To understand the evolution of genes, the data obtained from salmonids are compared against results from the Northern pike genome, a species that is basal to salmonids, but lacks the 4WGD (Rondeau et al. 2014) (Fig. 1). Orthology between salmonids and pike are based on high density linkage maps or chromosomal alignments published by Christensen et al. and Sutherland et al., summarized in Additional file 2 (Christensen et al. 2018b; Sutherland et al. 2016). For brown trout, the linkage groups presented by Leitwein and co-workers (Leitwein et al. 2017) do not match the chromosome numbers in the NCBI genome, so regional orthology is here based on blast match with region specific genes from other salmonids when this was informative.

We chose to define pseudogenes as those genes with internal stop codons. Sequences containing complete single domains were included in our phylogenetic analyses but may represent pseudogenes. One should keep in mind that although gene sequences are incomplete or pseudogenes in the genomes analyzed here, these genes may well be functional in other animals.

### DE lineage gene sequences

**Fig. 2:**
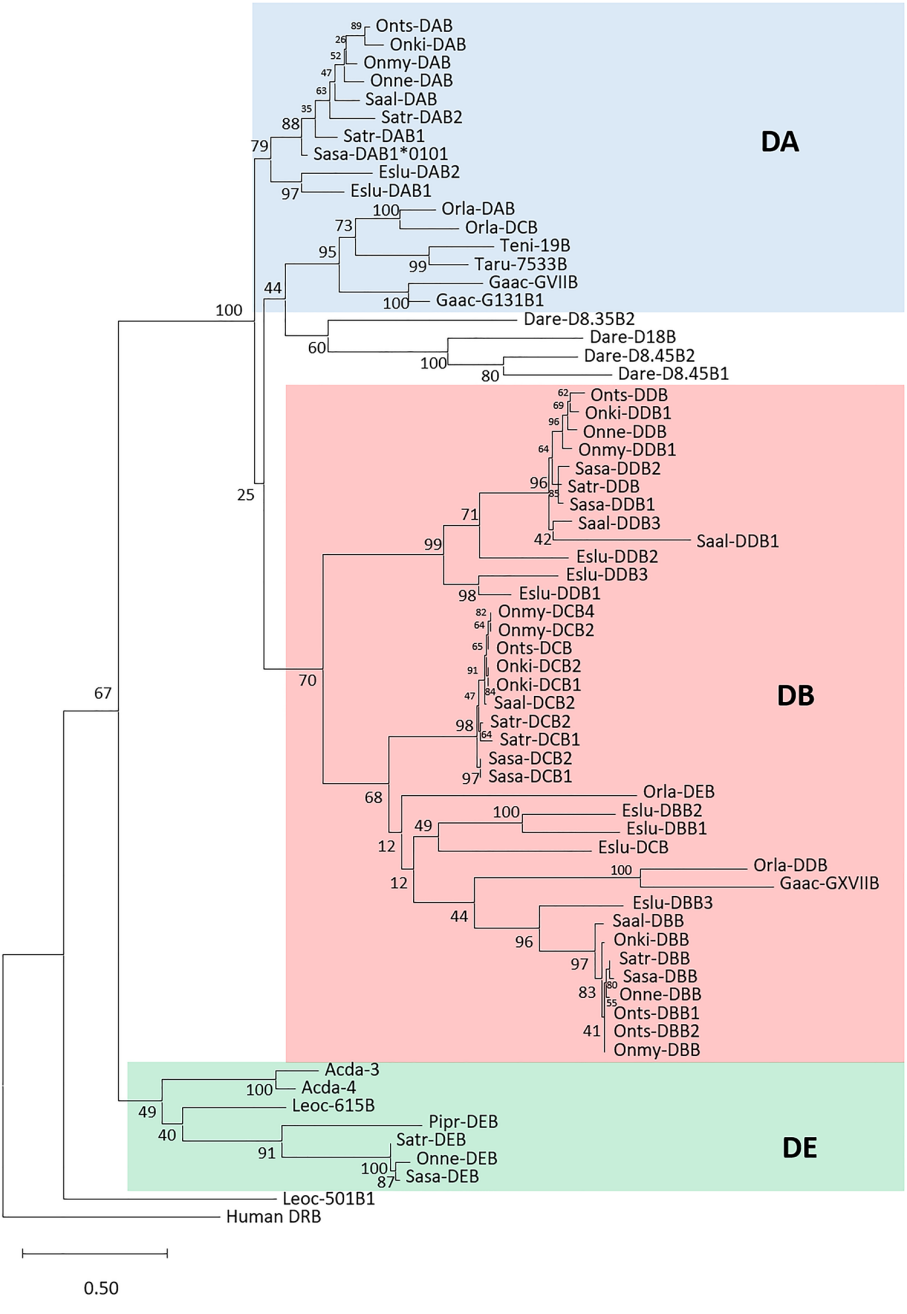
Phylogeny of deduced MHCII beta domain sequences. The evolutionary history was inferred by using the Maximum Likelihood method and Whelan And Goldman model with a bootstrap test of 100 replicates (Whelan and Goldman 2001). The tree with the highest log likelihood (− 9796.78) is shown. The percentage of trees in which the associated taxa clustered together is shown next to the branches. A discrete Gamma distribution was used to model evolutionary rate differences among sites (5 categories (+ G, parameter = 1.3808)]. The rate variation model allowed for some sites to be evolutionarily invariable ([+ I], 3.60% sites). The tree is drawn to scale, with branch lengths measured in the number of substitutions per site. This analysis involved 59 amino acid sequences. All positions with less than 95% site coverage were eliminated; i.e., fewer than 5% alignment gaps, missing data, and ambiguous bases were allowed at any position (partial deletion option). There were a total of 172 positions in the final dataset. DA, DB, and DE lineages are colored. The species abbreviations are Eslu for *Esox Lucius* (Northern pike), Sasa for *Salmo salar* (Atlantic salmon), Onmy for *Oncorhynchus mykiss* (rainbow trout), Onts for *Oncorhynchus tshawytscha* (chinook salmon), Onne for *Oncorhynchus nerka* (sockeye salmon), Onki for *Oncorhynchus kisutch* (coho salmon) and Saal for *Salvelinus alpinus*/*malma* (charr), Acda for *Acipenser dabryanus* (Dabry's sturgeon), Dare for *Dario rerio* (Zebrafish), Taru for *Takifugu rubipes* (Pufferfish), Teni for *Tetraodon nigroviridis* (Tetraodon), Gaac for *Gasterosteus aculeatus* (Three-spined stickleback), Leoc for *Lepisosteus oculatus* (Spotted gar), Orla for *Oryzias latipes* (Medaka), and Pipr for *Pimephales promelas* (Fathead minnow). The two Dabry’s sturgeon sequences are representatives of multiple DE sequence variants (Chen et al. 2020)

We previously identified DE lineage genes as the most ancient MHC class II lineage in teleosts, with DE lineage gene sequences also present in ray-finned paddlefish and sturgeons (Dijkstra et al. 2013). Atlantic salmon DE lineage genes (Sasa-DEA/-DEB, Sasa-DFA/-DFB), residing on chr.2 and the homeolog chr.5, are also surrounded by typical mammalian MHC region genes PHF1, RGL2, NOTCH, TAP1, and BRD2 (Horton et al. 2004), supporting this claim. Other salmonid DE lineage gene regions also contained genes syntenic to the human MHC region such as PHF1, RGL2, NOTCH, TAP1, and BRD2. Phylogenetically, the DE lineage sequences cluster at the base of all teleost MHCII sequences in all domain phylogenies (Fig. 2; Additional file 4), supporting their claim as the foundation for other MHCII genes in teleosts.

**Fig. 3:**
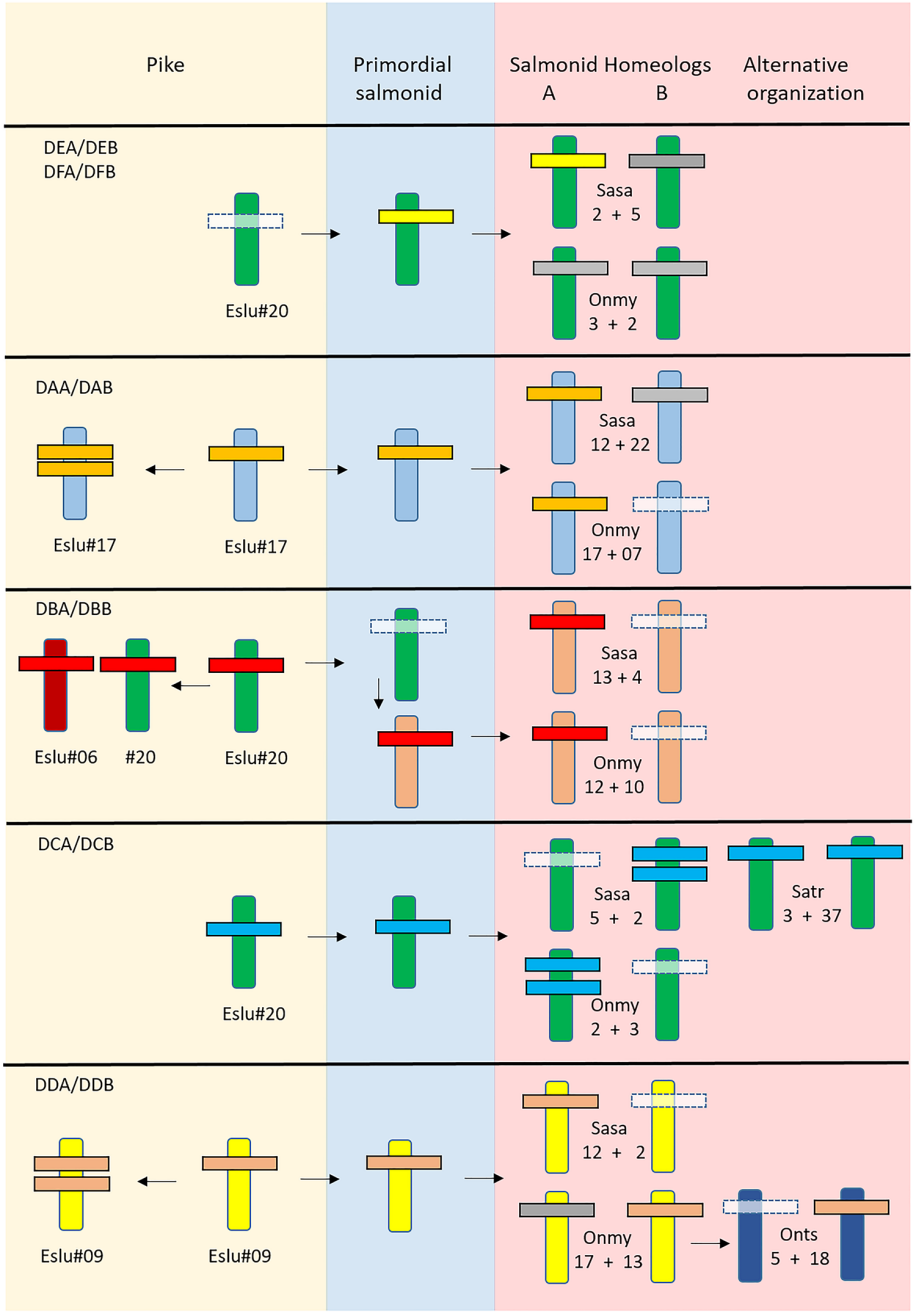
MHCII evolution from pike to salmonids. Schematic overview of how the MHCII genes evolved from Northern pike to a primordial salmonid to duplicate salmonid homeologs in addition to changes in Northern pike after it split from salmonids. Species are shown using abbreviations as follows: Eslu is *Esox lucius*, Sasa is *Salmo salar*, Satr is *Salmo trutta*, Onmy is *Oncorhynchus mykiss*, and Onts is *Oncorhynchus tshawytscha*. Atlantic salmon (Sasa) and rainbow trout (Onmy) are shown as representatives for the Salmo and Oncorhynchus lineages as many MHCII genes resided on unplaced scaffolds in charr and several salmonids. Homeolog chromosomes are shown as vertical bars using identical color and also with numbers, non-homeolog chromosomes using different colors. Each MHCII lineage mostly consisting of MHCII alpha and beta genes are shown using horizontal bars with different colors for each gene group. Lost genes are shown using transparent white bars, while grey bars define pseudogenes. Gene duplications are shown using two horizontal bars representing both single gen duplications as well as multiple gene duplications. Arrows indicate direction of evolution. Evolution in pike, a primordial salmonid, and salmonids are shaded yellow, blue, and red, respectively

We could not identify DE lineage orthologs in any of the five pike genome assemblies available in NCBI, so these genes have most likely been lost in Northern pike. The mammalian MHC region genes BDR2 and TAP1 reside on Northern pike chr.20, three Mb apart from the Eslu-DB/DC genes, but without traces of DE lineage genes (Additional file 3). In the remaining salmonids, we found DE lineage genes on both homeologs of Northern pike chr.20 (Fig. 3; Additional file 1, 2, 3). However, many of the salmonid DE lineage genes are pseudogenes similar to what we found for the Atlantic salmon *Sasa-DFA* and *Sasa-DFB* genes residing on chr.5 (Dijkstra et al. 2013). The pike and salmonid DE genes appear to be subject to ongoing deterioration suggesting, e.g., a possible loss of functional role or other selectional disadvantage in these species.

### DA lineage gene sequences

In teleosts, the DA lineage contains classical as well as non-classical MHCII genes and DA lineage sequences have been found in most teleosts studied so far (Dijkstra et al. 2013). Atlantic salmon and rainbow trout have only single classical DAA and a closely linked DAB gene belonging to this lineage. These two classical Atlantic salmon genes are linked to resistance against *Aeromonas salmonicida*, a bacteria causing the disease furunculosis (Grimholt et al. 2003; Kjoglum et al. 2008; Langefors et al. 2001; Lohm et al. 2002). Also, in *Salvelinus fontanalis*, the classical MHCII genes are linked to resistance against furunculosis, suggesting this is a common phenomenon for all salmonids (Croisetiere et al. 2008).

In pike, we found five MHCII gene sequences phylogenetically clustering with salmonid DA lineage sequences (Fig. 2; Additional file 1, 3-4). The pike *Eslu-DAA1*, *Eslu-DAA2*, *Eslu-DAA3*, *Eslu-DAB1*, and *Eslu-DAB2* genes are closely linked on chr.17 and only the *Eslu-DAA2* gene is a pseudogene. The functional status of the duplicate pike DA lineage genes remains to be established.

Atlantic salmon has MHCII genes and a gene fragment on both homeologs of pike chr.17 (Fig. 3; Additional file 1, 3). The two closely linked classical *Sasa-DAA* and *Sasa-DAB1* genes reside on chr.12, while a *Sasa-DAB2* pseudogene fragment resides on chr.22. Both Atlantic salmon *Sasa-DAB* regions on chr.12 and chr.22 share several syntenic genes with pike. Rainbow trout has *Onmy-DAA* and *Onmy-DAB* genes on chr.17, which is orthologous to pike chr.17 and Atlantic salmon chr.12 (Fig. 3). This trout chr.17 region also shares syntenic TFEB and TMEM183A genes with the DAA/DAB region on Northern pike chr.17. The rainbow trout chr.17 homeolog, chr.7, does not have any DAA/DAB remnants (data not shown). Brown trout has *Satr-DAA2* and *Satr-DAB2* genes on chr.36 in addition to duplicate *Satr-DAA1* and *Satr-DAB1* genes residing on an unplaced scaffold (Additional file 3), both regions with no genes syntenic with the Northern pike DAA/DAB region on chr.17. Unfortunately, coho, chinook, sockeye, brown trout, and charr all have DA lineage genes on unplaced scaffolds with no syntenic genes, providing no insight into their orthology with other pike and salmonid regions.

There are reports on the polymorphic nature of DAA and DAB genes for the salmonids Atlantic salmon, rainbow trout, coho salmon, brown trout, and charr (Consuegra et al. 2005; Croisetiere et al. 2008; Glamann 1995; Gomez et al. 2010; Hansen et al. 1999; Miller and Withler 1996; Shum et al. 2001; Stet et al. 2002; Wynne et al. 2007), while no such data exists for pike, sockeye, and chinook salmon. With single DAA and DAB genes, Atlantic salmon and rainbow trout have 42 and 22 DAB alleles registered in the IPD-MHC database today. The polymorphic nature of duplicate DAA/DAB genes in both brown trout as well as those of Northern pike needs further studies to define their classical nature.

Although we identified DA lineage genes from many other teleosts (Dijkstra et al. 2013) (Additional file 4), we were unable to define each individual gene as classical or non-classical due to lack of definite expressed match. The only non-salmonid teleost where classical MHCII genes have been defined is medaka (Bannai and Nonaka 2013). Also, in medaka, there is only one DAA and one closely linked DAB gene that encodes classical MHCII genes. The number of DA lineage genes in teleost species has a large span, where for instance tilapia has 18 gene sequences clustering with other DA lineage sequences while Atlantic cod has none (Dijkstra et al. 2013; Sato et al. 2012; Star et al. 2011). The span in number of MHCII genes may reflect functional diversification of how exogenous peptides are treated.

### DB group gene sequences

#### DBA and DBB gene sequences

MHCII sequences belonging to the DB group are represented by *Sasa-DBA*/*-DBB*, *Sasa-DCA*/*-DCB*, and *Sasa-DDA/*-DDB genes in Atlantic salmon. They are all defined as non-classical with low polymorphism and restricted tissue expression patterns (Harstad et al. 2008). We have no evidence showing their functional relevance, but *Sasa-DBB* and *Sasa-DDA* had high expression in spleen, *Sasa-DCA* was highly expressed in hind gut, while *Sasa-DBA* displayed low expression in all tissues.

**Fig. 4:**
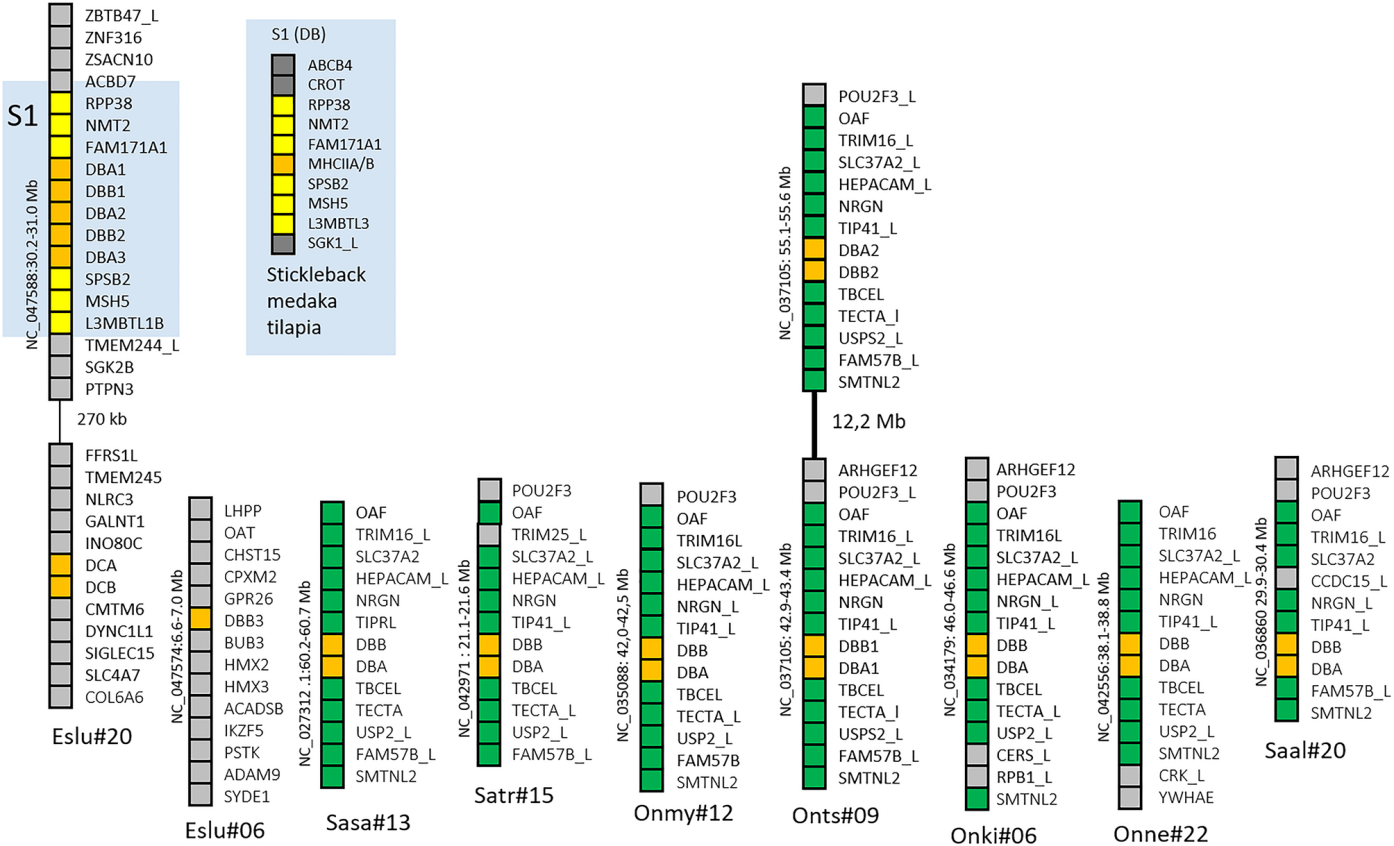
Genomic regions for DBA and DBB genes. Genomic regions with salmonid DB group genes compared against Northern pike DB region. Chromosomal location of each region is shown below as well as on the left hand side of each region. Gene boxes are color shaded as follows: orange boxes are MHCII genes, green boxes represents syntenic genes found between salmonids, yellow boxes are genes in Northern pike syntenic to the previously identified S1 synteny block found in medaka, stickleback, and tilapia (Dijkstra et al. 2013), while remaining genes are shown using grey boxes. The black line in Chinook salmon chromosome 9 (Onts#9) represents a regional gaps of 12 Mb. For species abbreviations, see legend to Fig. 2

Based on phylogenetic analysis, the previously identified *Sasa-DBA* and *Sasa-DBB* gene sequences have orthologs in the species analyzed (Additional file 1-2, 4). Northern pike has three Eslu-DBA and two Eslu-DBB genes on chr.20 in addition to an *Eslu-DBB3* gene on chr.6 (Fig. 4). In contrast, salmonids all have single DBA and DBB genes with the exception of chinook salmon that has duplicate DBA and DBB loci on chr.9. All pike and salmonid DBA and DBB gene sequences comply with our definition of bona fide genes.

There is no chromosomal orthology between pike chr.20 and salmonid chromosomes harboring DBA and DBB genes. Instead, salmonid DBA and DBB genes reside on chromosomes orthologous to pike chr.7 (Fig. 4; Additional file 2). For instance, Atlantic salmon *Sasa-DBA* and *Sasa-DBB* genes reside on chr.13, whereas orthologs to pike chr.20 are Atlantic salmon chr.2 and chr.5. A pike region on chr.7, showing synteny to the salmonid DBA-DBB regions, including the genes TECTA, TBCEL, TIPRL, and NRGN, does not contain MHCII genes (data not shown). The pike DBA/DBB genes then translocated from equivalents of pike chr.20 to an equivalent of pike chr.7 in a primordial salmonid, i.e., the period between the split from Northern pike and before salmonids emerged as separate species (Figs. 1 and 3). The additional *Eslu-DBB3* gene located in a region on pike chr.6 shares no syntenic genes with any salmonid MHCII regions, suggesting this gene translocated from pike chr.20 to pike chr.06 after pike split from the salmonid lineage.

The lack of orthology between pike and salmonid DBA/DBB regions is also visible in the genomic surroundings, where salmonid DBA and DBB genes are flanked by TIP41 and TBCEL while the pike Eslu-DBA and Eslu-DBB genes are flanked by SPSB2 and FAM171A1 (Fig. 4). In fact, the regional pike DBA and DBB genes match genes in the synteny 1 (S1) region of stickleback, tilapia, and medaka DB lineage genes (Dijkstra et al. 2013). Typical S1 region genes SGK1, L3MBTL3, MSH5, SPSB2, FAM171A1, NMT2, RPP38, CROT, and ABCB4 found in the above mentioned neoteleosts also embrace the pike Eslu-DBA/-DBA genes. This S1 region was one of three syntenic MHCII regions (S1–S3) previously found shared between neoteleosts only, where the syntenic regions S1 or S2 contained DB group genes, while the S3 region contained DA lineage genes. Equivalents of S2 and S3 synteny regions are not found in pike or in studied salmonids. Loss of S1 synteny in salmonids and lack of chromosomal orthology suggests that the chromosomal translocation from a pike chr.20 to an equivalent of pike chr.7 in a primordial salmonid thus abolished the regional S1 synteny in salmonids.

In phylogenies, the pike DBA gene sequences cluster with salmonid DBA sequences with convincing bootstrap support (Additional file 4). Phylogenies of pike DBB gene sequences display less convincing clustering with salmonid DBB sequences with the exception of the pike DBB3 gene sequence (Fig. 2).

#### DCA and DCB gene sequences

**Fig. 5:**
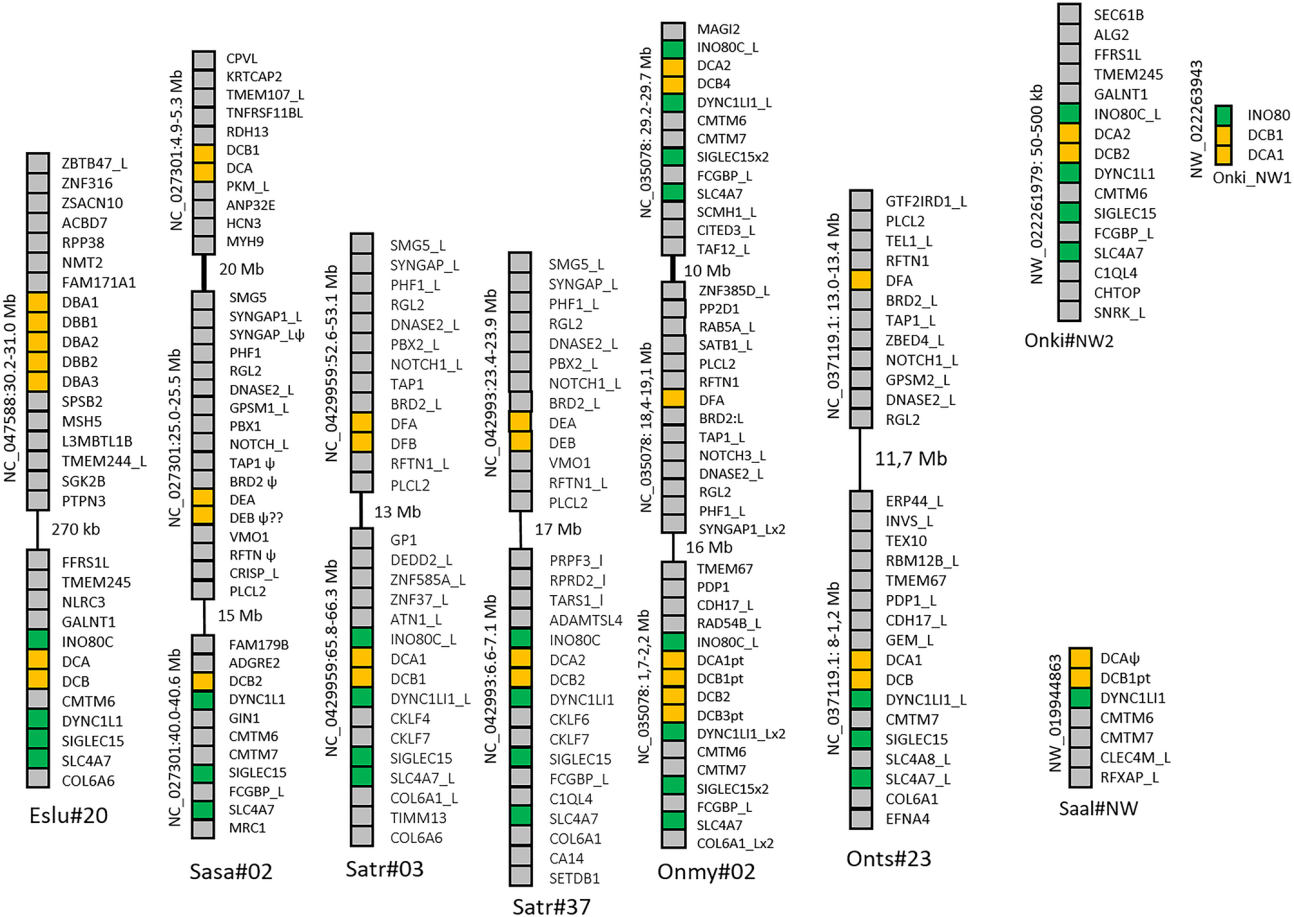
Genomic regions for DCA and DCB genes. Genomic regions with salmonid DC group genes compared against Northern pike DC region. Chromosomal location of each region is shown below as well as on the left-hand side of each region. Gene boxes are color shaded as follows: orange boxes are MHCII genes, green boxes represents syntenic genes found in Northern pike and salmonids, blue boxes are genes with synteny to the mammalian MHC region, while remaining genes are shown using grey boxes. Regional gaps are shown with black lines. Genomic scaffolds with a single MHCII gene without additional gene predictions not shown are Onne-DCA1 NW_021791902; Onne-DCA2ψ NW_021792314, Onne-DCBpt NW_021784836, Onmy-DCA3pt NW_018587944.1, Onmy-DCB5pt NW081528397.1, Onts-DCA2 NW_020138771.1, and Saal-DCB2 NW_019947682. For species abbreviations, see legend to Fig. 2

To understand more about the evolution of DB group sequences, we studied DCA and DCB genes. Northern pike has single *Eslu-DCA* and *Eslu-DCB* genes, while the remaining salmonids have from one to three DCA genes and one to five DCB genes (Fig. 5; Additional file 1). Eight of thirty genes are incomplete gene sequences, and seven of the salmonid DCA and DCB gene sequences reside as single genes on unplaced scaffolds. If these sequences are pseudogenes or genes in complex repeat regions making assembly difficult need further studies.

Northern pike has one *Eslu-DCA* and one *Eslu-DCB* gene residing on chr.20 flanked by INO80C and SIGLEC15, approximately 400 kb downstream of the Eslu-DBA and Eslu-DBB genes (Fig. 5). Previously identified Atlantic salmon *Sasa-DCA* and *Sasa-DCB1* genes (Harstad et al. 2008) reside on chr.2, a chromosome orthologous to pike chr.20 (Additional file 2), with no flanking genes shared with the Eslu-DC region. However, an additional *Sasa-DCB2* gene residing 35 Mb downstream is flanked by the syntenic DYNC1L1, SIGLEC15, and SLC4A7 genes. Rainbow trout has a DC gene duplication resembling Atlantic salmon also on a chromosome orthologous to pike chr.20, where both regions have flanking INO80C and SIGLEC genes. Brown trout, on the other hand, has DCA/DCB gene pairs on the two homeologs chr.3 and chr.37 based on the presence of DE lineage genes on both chromosomes. In brown trout, there is no sign of an additional duplicate gene on either chr.3 nor chr.37 in contrast to what is found in Atlantic salmon and in rainbow trout, suggesting the DCA/DCB genes were present in both homeologs in a primordial salmonid and then lost on one homeolog in Atlantic salmon and rainbow trout (Fig. 3). This is supported by looking at chromosomal orthology, where rainbow trout and chinook salmon have DCA and DCB genes on one chromosome orthologous to pike chr.20, while Atlantic salmon also has DC genes on a chromosome orthologous to pike chr.20, but on the other homeolog compared with rainbow trout and chinook salmon (Additional file 2). To explain this discrepancy between brown trout, Atlantic salmon and rainbow trout, the gene duplication found on Atlantic salmon chr.2 and rainbow trout chr.2 may have occurred separately in these two species.

In phylogenies, the pike DCA gene sequence clustering with salmonid DCA sequences is strongly supported (Additional file 4). In phylogenies of MHCIIB, both pike DBB1, pike DBB2, and pike DCB gene sequences show a more diffuse clustering to salmonid DBB and DCB sequences. The DBA/DBB to DCA/DCB gene duplication probably occurred in Northern pike, where the DBA/DBB genes translocated to a new chromosome in a primordial salmonid, while the DCA/DCB genes remained. The whole genome duplication in salmonids then provided the primordial salmonid with dual DCA/DCB copies conserved in brown trout. Other species such as Atlantic salmon and rainbow trout lost the duplicate on opposite homeologs but both these species added another translocation of DCA/DCB genes to their chosen homeolog. In summary, the DBA/DBB and DCA/DCB genes are prime examples of the diversity in MHCII duplications and chromosomal translocations.

#### DDA and DDB gene sequences

**Fig. 6:**
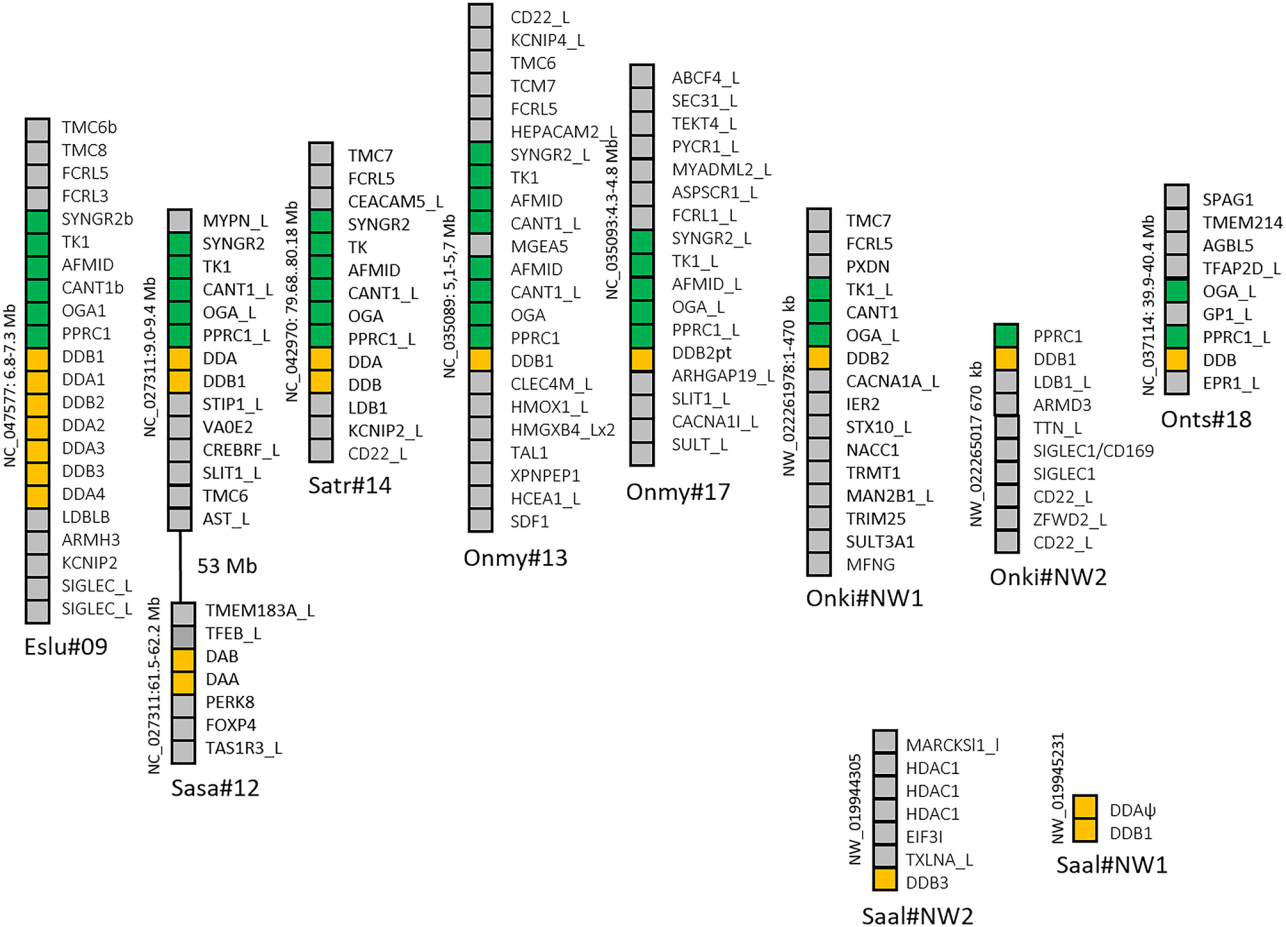
Genomic regions for DDA and DDB genes. Genomic regions with salmonid DD group genes compared against Northern pike DD region. Chromosomal location of each region is shown below as well as on the left hand side of each region. Gene boxes are color shaded as follows: orange boxes are MHCII genes, green boxes represents syntenic genes found in Northern pike and salmonids, while remaining genes are shown using grey boxes. Regional gap is shown with black line. Not shown genomic unplaced scaffolds with one single MHCII gene without additional gene predictions are *Saal-DDB2ψ* NW_019952256 and *Onne-DDB* NW_021797582. We found no DDA genes in Rainbow trout, Coho salmon, Chinook salmon, and Sockeye salmon. For species abbreviations, see legend to Fig. 2

The final Atlantic salmon DB group genes are the *Sasa-DDA* and *Sasa-DDB* genes (Dijkstra et al. 2013; Harstad et al. 2008). In Northern pike we found seven orthologs to the Atlantic salmon *Sasa-DDA* and *Sasa-DDB* gene sequences here denoted *Eslu-DDA1-4* and *Eslu-DDB1-3* (Fig. 6; Additional files 1 & 4). All seem like bona fide genes closely linked on pike chr.9, with the regional syntenic TK1, CANT1, OGA, and PPRC1 genes compared with the Atlantic salmon *Sasa-DDA*/*-DDB* region on chr.12 (Fig. 6; Additional file 2). The Atlantic salmon chr.2, a chr.12 homeolog, does not contain any DDA/DDB gene sequences.

We found DDB genes in all salmonids where both coho salmon and charr have duplicate DDB genes on unplaced scaffolds, while the remaining salmonids have single DDB genes (Fig. 6). In contrast, DDA genes were lacking in several salmonid species. We did not find the DDA gene in the rainbow trout genome from a Swanson river animal, but this gene was present in the Arlee animal genome (data not shown) and we also found a matching rainbow trout TSA in NCBI (GBTD01042060.1). Also, other Oncorhynchus species, i.e., coho, Chinook, and sockeye salmon genomes, lack the DDA gene, suggesting this gene is deteriorating in Oncorhynchus species. Supporting this hypothesis is the fact that the charr *Saal-DDA* and *Saal-DDB2* genes both have characteristics of being pseudogenes. In Atlantic salmon we found high *Sasa-DDA* expression in hindgut (Harstad et al. 2008) suggesting a yet unknown function at least in this species. The expression patterns of the duplicated pike Eslu-DDA and Eslu-DDB genes remain to be established.

Northern pike DDA/DDB genes reside on chr.9, with orthology to Atlantic salmon chr.12, harboring the *Sasa-DDA* and *Sasa-DDB* genes (Fig. 6; Additional file 2). Rainbow trout has one bona fide *Onmy-DDB1* gene on chr.13, and also a *Onmy-DDB2* gene fragment on chr.17, also orthologs of pike chr.9. However, the Atlantic salmon chr.12 is not a homeolog of rainbow trout chr.13. So the primordial salmonid had DDA/DDB genes on both homeologs, where different homeologs have lost the DDA/DDB gene function in Salmo vs Oncorhynchus species (Fig. 3). Chinook salmon, on the other hand, has a DDB gene on chr.18, which is not orthologous to either pike chr.9, Atlantic salmon chr.12, or rainbow trout chr.13. This suggests that both DDA/DDB homeologs on chr.02 and 32 have been lost in chinook salmon, while the DDB gene has translocated to chr.18 and the DDA gene has been lost. Coho salmon, sockeye salmon, and Salvelinus have their DDA/DDB genes on unplaced scaffolds providing no further clue as to the evolutionary history of these genes.

Phylogenies do not provide much clarification as to the evolutionary history of DDA/DDB genes in pike and salmonids. Both pike and salmonid DDB sequences cluster with DBB and DCB sequences, suggesting these genes are older duplicates of DBB/DCB genes (Fig. 2). But this view is not supported by MHCIIA phylogenies where they cluster either with DA lineage sequences or form a separate cluster alongside DA and DB/DC sequences (Additional file 4). We have not found DDA/DDB gene sequences from any other teleost species so we believe they originated at the base of the pike/ salmonid branch.

### Salmonid MHCII evolution in a teleost perspective

All current and previous studies show the DE lineage as the ancestor of other teleost MHCII lineage/ group sequences (Fig. 2; Additional file 4) (Dijkstra et al. 2013). One exception is the spotted gar sequences Leoc-501A1/-B1 sequences that are even basal to DE clade. This could imply that they are remnants of an even older lineage lost in other species. However, there are only DE lineage genes in paddlefish and sturgeons, with no MHCII sequences of any other groups or lineages. In sturgeon these DE lineage genes may function as classical MHCII molecules supported by multiple transcribed sturgeon DE lineage sequences defined as alleles (Chen et al. 2020).

DA lineage and DB group genes are diverged duplicates originating from the DE lineage where the evolutionary relationship between DA lineage and DB group sequences is unclear. Classical MHCII sequences defined in medaka and Atlantic salmon belong to the DA lineage (Bannai and Nonaka 2013; Dijkstra et al. 2013). DA lineage sequences are present in both pike and salmonids, but their polymorphic nature in pike, brown trout, Chinook, and sockeye salmon need investigation before they can be defined as classical genes (Fig. 2; Additional file 4). Atlantic salmon and pike DBA/DBB and DCA/DCB sequences cluster with medaka and stickleback DB group sequences, while pike and salmonid DDA/DDB gene sequences may be unique to pike and salmonids.

There is increasing support for the unstable nature of MHCII in teleosts. First of all the MHCII was most likely translocated out of the MHC region in a primordial teleost as both MHCI and MHCII are linked in spotted gar. Lack of synteny between zebrafish, Atlantic salmon and neoteleosts (Dijkstra et al. 2013) also suggested MHCII instability with species specific chromosomal translocations. This is further supported by finding one of the neoteleost syntenic regions in pike, but then seeing it lost en route to salmonids.

## Conclusion

We initiated this study to understand how the fourth whole genome duplication had affected MHCII genes in salmonids using Northern pike as a reference. Fate of genes originating from whole genome duplications is either silencing, sub-functionalization, or neofunctionalization. In the majority of cases salmonid duplicate genes have died or are dying on one homeolog, where genes have been silenced on different homeologs in the Salmo and Oncorhynchus lineages for DCA/DCB and DDA/DDB gene duplicates. Gene translocations are also apparent, where for instance the DBA/DBB genes have translocated to a different chromosome in a primordial salmonid prior to the whole genome duplication. Such a translocation has also occurred in chinook salmon where the DDA/DDB genes have translocated to a different chromosome compared with the Oncorhynchus relatives. Unique single gene duplications have occurred in some species where in particular the DB group has expanded considerably in Northern pike and rainbow trout. The DB group genes are retained as bona fide genes in pike, while many are only partial gene sequences in trout. Northern pike also has potentially duplicate classical DAA/DAB genes, while in brown trout one of the DAA/DAB gene pairs is dominant. In salmonids, both Atlantic salmon as well as rainbow trout have duplicated their DCA/DCB genes. The overall picture is that MHCII genes have mostly reverted to single copy genes, while some are tolerated as gene duplicates. Further studies are needed to understand the biological role of teleost MHCII genes, in particular the non-classical genes.

## Electronic supplementary material

Below is the link to the electronic supplementary material.

Supplementary file1 (PDF 887 kb)

Supplementary file2 (PDF 478 kb)

Supplementary file3 (PDF 209 kb)

Supplementary file4 (PDF 666 kb)

## Data Availability

All data generated are presented in main text or supplementary files.
