# Supplementary material for: Fate of MHCII in salmonids following 4WGD
Source: Immunogenetics. 2020 Nov 23;73(1):79–91. doi: 10.1007/s00251-020-01190-6 (PMC7862078; doi:10.1007/s00251-020-01190-6)
Supplement: Supplementary file 1 — Supplementary file1 (PDF 887 kb) [file 251_2020_1190_MOESM1_ESM.pdf]

Additional file 1 (AF1). Deduced MHCII amino acid sequence

		Page
AF1.0	Table of MHCII alpha and MHCII beta genes, accession numbers and genomic location from pike and salmonids	2
	Deduced amino acid sequences	
AF1.1	Atlantic salmon (*Salmo salar*)	5
AF1.2	Brown trout (*Salmo trutta*)	6
AF1.3	Rainbow trout (*Oncorhynchus mykiss*)	7
AF1.4	Sockeye salmon (*Oncorhynchus nerka*)	9
AF1.5	Coho salmon (*Oncorhynchus kisutch*)	10
AF1.6	Chinook salmon (*Oncorhynchus tshawytscha*)	11
AF1.7	Charr (*Salvelinus alpinus/ malma*)	12
AF1.8	Northern pike (*Esox Lucius*)	13
AF1.9	Sequences used in phylogenies from other species	15
	Spotted gar (*Lepisosteus oculatus*	15
	Tetraodon (*Tetraodon nigroviridis*)	16
	Fugu (*Takifugu rubripes*)	16
	Medaka (*Oryzias latipes*)	17
	Stickleback (*Gasterosteus aculeatus*)	17
	Zebrafish (*Danio rerio*)	18
	Darby's sturgeon (*Acipenser dabryanus*)	19
	Fathead minnow (Pimephales promelas)	19

Deduced amino acid sequences from *Salvelinus malma* (Saal), *Oncornhynchus nerka* (Onne), *Oncorhynchus kisutch* (Onki), *Oncorhynchus tshawytscha* (Onts), *Oncorhynchus mykiss* (Onmy), *Salmo trutta* (Satr), *Salmo salar* (Sasa) and *Esox Lucius* (Eslu) originating from the genomes described in main text Material and Methods. When available, the GenBank protein accession number and chromosomal location is provided for each sequence. Gene with internal stop codon has -ps included in gene name while partial genes containing a complete single domain or parts of domains are given a -pt extension.

AF1.0 Table of MHCII alpha and MHCII beta sequences from pike and salmonids

MHCIIA genes	Accession #	Chr.	Genomic location
Eslu_DAA1	NP_001290803.1	chr.17	NC_047585.1:25.160.320-25.165.305
Eslu_DAA2ψ	XP_034143102.1	chr.17	NC_047585.1:25.223.821-25.225.006
Eslu_DAA3	XP_034143470.1	chr.17	NC_047585.1:25.303.871-25.315.116
Eslu_DBA1	XP_010883054.2	chr.20	NC_047588.1:30.293.930-30.298.643
Eslu_DBA2	XP_034144659.1	chr.20	NC_047588.1:30.310.087-30.314.003
Eslu_DBA3	XP_019896238.2	chr.20	NC_047588.1:30.322.856-30.325.597
Eslu_DCA	XP_019897056.2	chr.20	NC_047588.1:30.854.142-30.855.420
Eslu_DDA1	XP_028978174.2	chr.09	NC_047577.1:7.021.282-7.025.210
Eslu_DDA2	XP_028978025.2	chr.09	NC_047577.1:7.044.026-7.054.127
Eslu_DDA3	XP_019905383.2	chr.09	NC_047577.1:7.061.743-7.066.269
Eslu_DDA4	XP_010864729.3	chr.09	NC_047577.1:7.077.058-7.083.387
Onki_DAA	XP_031673251.1	chr.01	NC_034174.2:30.733.108-30.738.210
Onki_DBA	XP_020340180.1	chr.06	NC_034179.2:46.329.716-46.335.674
Onki_DCA1	XP_031675123.1		NW_022263943.1:7.643-10.265
Onki_DCA2	XP_031667867.1		NW_022261979.1:311.232-314.542
Onki_DEA	XP_031657800.1	chr.02	NC_034175.2:19.663.235-19.668.779
Onki_DFA	No	chr.13	NC_034186.2:59.583.236-
Onmy_DAA	XP_021425091.1	chr.17	NC_035093.1:49.670.799--49.671.350
Onmy_DBA	XP_021479589.1	chr.12	NC_035088.1:42.256.089-42.261.363
Onmy_DCA1	No	chr.02	NC_035078:1.949.498-1.950.031
Onmy_DCA2	XP_021419620.1	chr.02	NC_035078:29.471.783-29.473.031
Onmy_DCA3	XP_021450880.1		NW_018587944.1:529-1.062
Onmy_DDA	GBTD01042060.1		no genomic match
Onmy_DEA	No	chr.03	NC_035079.1:28.508.845-28.509.058
Onmy_DFA	No	chr.02	NC_035078.1:18.600.094-18.601.625
Onne_DAA	XP_029508585.1		NW_021807294.1:41.778-44.505
Onne_DBA	XP_029483109.1	chr.22	NC_042556.1:38.336.566-38.342.193
Onne_DCA	XP_029503552.1		NW_021791902.1:24-1.239
Onts_DAA	XP_024264231.1		NW_020130363.1:60.941-65.768
Onts_DBA	XP_024288155.1	chr.09	NC_037105.1:55.361.845-55.367.850
Onts_DCA1	XP_024267460.1		NW_020138771.1:20-1.199
Onts_DCA2	XP_024287887.1	chr.09	NC_037105.1:43.135.612-43.141.617
Onts_DCA3	XP_024241033.1	chr.23	NC_037119.1:991.276-992.667
Onts_DEA	No	chr.03	NC_037099.1:16.108.478-16.110.728
Onts-DFA	No	chr.23	NC_037119.1:13.088.214-13.090.095
Saal_DAA	XP_023999496.1		NW_019945608:1634-3556
Saal_DBA	XP_023866627.1	chr.20	NC_036860.1:30.150.199-30.155.979
Saal_DCA	No		NW_019944863.1:7.697-8.641
Saal_DDA	XP_023999181.1		NW_019945231:17.363-22.556
Saal_DEA	No	chr.35	NC_036874.1:9.075.676-9.078.023
Saal_DFA	No	chr.6.1	NC_036845:13.836.333-13.838.339
Sasa_DAA*0101	L77086.1	chr.12	NC_027311.1:61.701.375-61.703.967

Sasa_DBA	EG757342,EU008541	chr.13	NC_027312.1:60.436.490-60.443.021
Sasa_DCA	DW549478,DY704572	chr.02	NC_027301.1:5.092.612-5.094.817
Sasa_DDA	DW557800	chr.12	NC_027311.1:9.215.607-9.218.392
Sasa_DEA	KC316032	chr.02	NC_027301.1:25.249.180-25.251.518
Sasa_DFAps	XP_014056167.1	chr.05	NC_027304.1:55.568.279-55.570.111
Satr_DAA1	XP_029600114.1		NW_021822588.1:3.641-5.620
Satr_DAA2	XP_029591961.1	chr.36	NC_042992.1:504.310-507.170
Satr_DBA	XP_029546517.1	chr.15	NC_042971.1:21.333.634-21.340.327
Satr_DCA1	No	chr.03	NC_042959.1:66.026.793-66.028.716
Satr_DCA2	No	chr.37	NC_042993.1:6.816.058
Satr_DDA	XP_029545631.1	chr.14	NC_042970.1:79.981.941-79.984.210
Satr_DEA	No	chr.37	NC_042993.1:23.645.472-23.647.788
Satr_DFA	No	chr.03	NC_042959.1:52.808.926-52.810.975
**MHCIIB genes**	**Accession #**	**chr.**	**Genomic location**
Eslu_DAB1	XP_034143025.1	chr.17	NC_047585.1:25.147.505-25.152.712
Eslu_DAB2	XP_034143218.1	chr.17	NC_047585.1:25.335.671-25.342.225
Eslu_DBB1	NP_001290706.1	chr.20	NC_047588.1:30.301.515-30.308.996
Eslu_DBB2	XP_010883050.2	chr.20	NC_047588.1:30.314.683-30.320.727
Eslu_DCB	XP_034144799.1	chr.20	NC_047588.1:30.855.505-30.860.622
Eslu_DBB3	XP_010867200.1	chr.06	NC_047574.1:6.834.048-6.839.218
Eslu_DDB1	XP_010870891.2	chr.09	NC_047577.1:6.996.793-7.014.272
Eslu_DDB2	XP_028978022.2	chr.09	NC_047577.1:7.035.721-7.040.359
Eslu_DDB3	XP_012990668.2	chr.09	NC_047577.1:7.068.106-7.075.388
Onki_DAB	XP_031673186.1	chr.01	NC_034174.2:30.740.885-30.746.625
Onki_DBB	XP_020339901.2	chr.06	NC_034179.2:46.340.511-46.343.391
Onki_DCB1	XP_031675120.1		NW_022263943.1:2.007-7.643
Onki_DCB2	XP_031667866.1		NW_022261979.1:314.625-319.616
Onki_DDB1	XP_031676050.1		NW_022265017.1:30.916-38.225
Onki_DDB2	XP_031666909.1		NW_022261978.1:2.892.149-2.893.573
Onmy_DAB	XP_021425090.1	chr.17	NC_035093.1:49.644.190-49.668.113
Onmy_DBB	XP_021479591.1	chr.12	NC_035088.1:42.266.576-42.269.097
Onmy_DCB1	No	chr.02	NC_035078.1:1.939.969-1.940.217
Onmy_DCB2	XP_021476352.1	chr.02	NC_035078.1:1.942.531-1.943.589
Onmy_DCB3	No	chr.02	NC_035078.1:1.944.223-1.944.501
Onmy_DCB4	XP_021419604.1	chr.02	NC_035078.1:29.466.615-29.469.923
Onmy_DCB5	No		NW_018528397.1:1299-1577
Onmy_DDB1	XP_021412280.1	chr.13	NC_035089.1:5.368.697-5.370.479,
Onmy_DDB2	No	chr.17	NC_035093.1:4.571.316-4.572.534
Onmy_DEB	XP_021449969.1	chr.03	NC_035079.1:28.510.176???28.511.183,
Onne_DAB	XP_029508586.1		NW_021807294.1:46.283-52.355
Onne_DBB	XP_029483107.1	chr.22	NC_042556.1:38.326.373-38.330.559
Onne_DCB	XP_029500393.1		NW_021784836.1:4-1.153)
Onne_DDB	XP_029505744.1		NW_021797582.1:351-1498

Onne_DEB	XP_029481671.1	chr.03	NC_042537.1:11.058.502-11.059.845
Onts_DAB	XP_024264230.1		NW_020130363.1:54.045-59.238
Onts_DBB1	No	chr.09	NC_037105.1:43.147.513-43.149.018
Onts_DBB2	XP_024288156.1	chr.09	nNC_037105.1:55.372.711-55.376.082
Onts_DCB	XP_024241171.1	chr.23	NC_037119.1:986.253-990.499
Onts_DDB	No	chr.18	NC_037114.1:40.224.963-40.227.299
Onts_DEB	XP_024251641.1	chr.03	NC_037099.1:16.111.442-16.115.521
Saal_DAB	XP_023999497.1		NW_019945608:6748-11843
Saal_DBB	XP_023868529.1	chr.20	NC_036860.1:30.142.406-30.145.022
Saal_DCB1	XP_023998766.1		NW_019944863.1:12.805-13.947
Saal_DCB2	XP_024000748.1		NW_019947682:21.354-23.308
Saal_DDB1	XP_023999181.1		NW_019945231:30.347-33.914
Saal_DDB2	XP_024001573.1		NW_019952256:1.709-3.388
Saal_DDB3	No		NW_019944305.1:90.396-105.291
Saal_DEB	No	chr.35	NC_036874.1:9.078.877-9.079.056
Saal_DFB	No	chr.6.1	NC_036845:13.838.788-13.839.510
Sasa_DAB*0101	CAA49726	chr.12	NC_027311.1:61.693.947-61.699.457
Sasa_DBB	DY726096, EU008541	chr.13	NC_027312.1:60.424.988-60.429.008
Sasa_DCB1	KC316031	chr.02	NC_027301.1:5.088.71-5.092.439
Sasa_DCB2	XP_014028990.1	chr.02	NC_027301.1:40.319.469-40.323.752
Sasa_DDB1	No	chr.12	NC_027311.1:9.234.346-9.240.793
Sasa_DDB2	XP_014039502.1		NW_012348659.1:10.114-11.741
Sasa_DEB	KC316036	chr.02	CM003280.1:25.252.264-25.253.576
Sasa_DFBps	No	chr.05	NC_027304.1:55.566.855-55.567.573
Satr_DAB1	XP_029591960.1	chr.36	NC_042992.1:498.192-502.800
Satr_DAB2	XP_029600113.1		NW_021822588:9.176-13.055
Satr_DBB	XP_029546518.1	chr.15	NC_042971.1:21.322.740-21.326.263
Satr_DCB1	XP_029601215.1	chr.03	NC_042959.1:66.032.511-66.033.418
Satr_DCB2	XP_029594157.1	chr.37	NC_042993.1:6.809.351-6.814.933
Satr_DDB	XP_029545630.1	chr.14	NC_042970.1:79.922.139-79.957.834
Satr_DEB	XP_029593400.1	chr.37	NC_042993.1:23.647.912-23.651.646
Satr_DFB	No	chr.03	NC_042959.1:52.808.461-52.807.743

No accession number means they are manually predicted.

AF1.1 Atlantic salmon (*Salmo salar*; Sasa)MHCI sequences

```
>Sasa-DAA*0101 chr.12 NC_027311.1:61.701.375-61.703.967 AAL40122.1
MKTSVIVLILCWQVYAEHKVLHIDLVITGCSDSDGLDMYGLDGEEMWYADFNKQEGVVAL
PPFADPFTFPGFYEQAVGNQGVCKGNLAKCIKAYKNPEEKIDPPHSSIYPRDDVDLGVEN
TLICHVSGFFPAPVRVRWTRNNQNLTEGVRLSTPYPNADFTLNQFSSLPFTPEEGDIYGC
TVEHKGLAEPLTRIWEPEVIQPSVGPDVFCGVGLTLGLLGVAAGTFFLIKGNQCN
>Sasa-DAB1*0101 chr.12 NC_027311.1:61.693.947-61.699.457 XP_013988542
MSMSIFCVSLTLVLSIFSGTDGYFEQVVRQCRYSSKDLQGIEFIDSYVFNKAEYIRFNST
VGKFVGYTELGVKNAEAWNSDAAVLAVERGELERYCKHNADLHYSTILDKTVEPHVRLSS
VAPPSGRHPAMLMCSAYDFYPKPIRVTWLRDGREVKSDVTSTEELANGDWYYQIHSHLEY
TPRSGEKISCMVEHISLTEPMVYHWDPSLPEAERNKIAIGASGLVLGAILALAGLIYYKK
KSSGVL
>Sasa-DAB2ps chr.22 NC_027321.1: 25.326.916-25.327.147
VEPHVRMSSMTPPSGRHPAMLMCSAYDFYPKPIRLTFIRNEELGDGDWYYQIHSHLEYTP
TSEEKISCMLKH
>Sasa-DBA chr.13 NC_027312.1:60.436.490-60.443.021 XP_013992518.
MSFEMNYSVIILILTGAVCTSAEIHHEIHFIFGCFESSDPAVGLEIDGDEVFYGDFNKNS
NTCLIADVVFTLPKFISITPEDKERACEYATISRVWCKDCIAWGKQSEPKIPKIKDAPES
TIYPRDEVELGVENTLICFVNDFFPPPVKVYWTKNEMEVTEGLSLSRYYPNKDGTFHQFS
SLSFTPQKEDVYICAVAHTALKEPKTREYKVSGSSAGPGPAVFCGVGLTLGLLGVATGIF
LIYKGKRATESQE
>Sasa-DBB chr.13 NC_027312.1:60.424.988-60.429.008 ABX44766.1
MYVLNCFSIHLLLLFSSLSEVVDSSDEDFAHDDAWCRFSSRDLHNMEYILEHHFNKILVA
QYNSTTERWTGYTAWGVISAEKWNEDPDEIPRRRTDMGVLCKPYANRIYNATEMFMVEPN
VTLRLEGPSSDSSLVCSVHFFYPKHIRVTWLRNGEEVTSDVTSTDVLANGLWSYQIQSYL
KYTPTTGERITCMVEHISQTEPKLYYWDPSLPKSEKNKIVIGVCGLLLGVVFVVAGLIYW
AKSTGRLLGLIGERDYGTCD
>Sasa-DCA chr.02 NC_027301.1:5.092.612-5.094.817 XP_014005526.1
MNLSVAIVVLTAVVCTSAEIPHETVYVLGCLEKTKVKAEAELQLDGEEVVYADFQSGQEV
WTLPEFLGPFSSSTVRNFYKNAVKGRRLCRDALALWIFEEKSPPEVKDAPESTIYPRAEE
ELGVENTLICFANHFYPPPVKVNWTKNGLEVTEGTSLSRYYPNEDGTFHQFSSLSFTPQE
GDVYGCTVKHTALEDPKTRFWEYEVREVSGSSAGPAVFCGVGLTLGLLGVATGTFLYVKG
QQFN
>Sasa-DCB1 chr.02 NC_027301.1:5.088.71-5.092.439 XP_014005512.1
MSVLNLSSIHLLLLFSSLSGVDGYFGHFEMRCRFSSEDPRDIEYLLQVYGNKKLLGQYNS
TTEKCTVYTQWMKNFTETACKGPAFLSERREEMNKYCSSNVPVVYGYLLDKAVEPYIRLR
SVESFSTRHPAMLVCSAYDFYPKPIRVTWLRDGQEVTSNVTSTEELVNGDWTYQIHSHLE
YTPTPGERITCMVEHFSLTEPKLYDWDPSLPGPERNKMVIGACGLLLGVVFIAAGLIYYR
KKSTEGRVLEPTMALPESYGTI
>Sasa-DCB2 chr.02 NC_027301.1:40.319.469-40.323.752 XP_014028990.1
MSVLNLSSIHLLLLFSSLSGVDGYFGHFEMRCRFSSEDPRDIEYLLQVYGNKKLLGQYNS
TTEKCTVYTQWMKNFTETACKGPAFLSERREEMNKYCSSNVPVVYGYLLDKAVEPYIRLR
SVESFSTRHPAMLVCSAYDFYPKPIRVTWLRDGQEVTSNVTSTEELVNGDWTYQIHSHLE
YTPTPGERITCMVEHFSLTEPKLYDWDPSLPGPERNKMVIGACGLLLGVVFIAAGLIYYR
KKSTGEETPAILCQNNQILFKSKFE
>Sasa-DDA chr.12 NC_027311.1:9.215.607-9.218.392 XP_013985893.1
MMALVFMILLFMLSSTQGQEQHVFNVINTRSETEEFNMTIVVDDNEYLHVYLNNKEGVVT
LPEWGNYVDCPICVRVAESRRADLNNDIKLFNLETPEAKVPPEIKLYAKDEVKLGINNSL
VCFVNNFFPPPVQVKWTKNDENVPKGVKVGQYATNSDYTFYRFSTLTFEPQEGDIYTCIV
DHTALDEPLTRTWEFEVPPRASVGPAVFCGLGLTLGLLGVATGTFFLVKGTQCQ
>Sasa-DDB1 chr.12 NC_027311.1:9.234.346-9.240.793 XP_013985890.1
MFSFFCPTDGHFTTTLDLCYYRGEDPHDVEYIWRAISDMVKVMEYNSTLNRFTGYTPIGI
HKAEQFNADPVVLASFQTPLYFFCKYYGALAYKAGLNSNVEPSVHLRSMTPHSDRHPSML
TCSAYKFYPKQIRVTWLRNGQEVTSNMTSSEELANGDWHHQIHSYLEYTPTPGEKISCMV
EHASFTEPKILHWDMSLPESGRNKIAIGASGLVLGVVFAAAGLLYYNRRKTTGGGGRELV
PTSHPSQ
>Sasa-DDB2 NW_012348659.1:10.114-11.741 corrected XP_014039502.1
VFMHSCFFSPDGHFITTLNLCYYRGEDPHDVEYIWRAISDMVKVMEYNSRFTGYTPIGIH
KAEQFNADPVVLASFQTPLHFFCKYYGALAYKAGLNSNVENKRILSHRSMTPHSDRHPSM
LTCSAYKFYPKQIRVTWLRNGQEVTSNMTSSEELANGDWHYQIHSYLEYTPTPGEKISCM
VEHASFTEPNILHWDMSLPESGRNKIAIGASGLVLGVVFAAAGLLYYNRRKTTGGGGE
>Sasa-DEA chr.02 NC_027301.1:25.249.180-25.251.518 No pred.
MGCRVFLAFILGVCLLSHCQSKHLLRFLTFCQKNVPSDEEYDVEFDGDELFYVDSITYQV
ERRLSEFAQQWTPDPGLPHEVYVSLGTCQYNIPRCIVGEKSPPEAIEVPTSHIYSQREVE
LGVPNTLICRVSDFHPTPVDVTWTRNEQPVAERTIIQTQYYSNEDFSFRIFSYLSITPQE
GDIYSCSVGHVSLQEPLTRIWEVEVHTDHQTVETAVCVGGVTLGVVGVATGVWFIKKAKR
SGWALRT
```

```
>Sasa-DEB chr.02 NC_027301.1: 25.252.264-25.253.576 No pred.
MALCWVYWMAGLSVIQTWATPAGGYQFQGIVDCEYDDTIDNMIYFVKNIFDQKLTTIYDS
RVQKYVGFGEFGIRNADRYNSQAWKMAIRKAEVETICRYSAIFFKLSTLERIVPPIVKVR
LTKPSRYGELSMLECSVLGFYPQEVRVSWLRDGLETTTAVTSTDTLANGDWSYQLHSYLE
FRPQRGESVSCMVEHPSLDEPLEVVWDTSGLDAKWFKMAIGVCSLFIGVAMAIGGGVYYW
WKNRSGFRRVNR
>Sasa-DFAps chr.05 NC_027304.1:55.568.279-55.570.111 XP_014056167.1
MGCRVFLALILGVCLLSHCQSKHLLRFLTFCQKNITHKEYDVEFEDELFYVDPMTYRVERR
LSAFAQQWTRDPGLPHEVNVSLGTCQYNIPCCIVGENEAPTSHIYSQREVELGDPNTLICR
VSDFHPTSVDVTWTRNEQPVGEGTVSLTQYYSNENFSFRIFSYLSITPQEGDIYSCSVGHV
SLQEPLTRIWEVEVHTDHQTVETAVCVGGVTLGVVGVATKKAKRSGWALRT
>Sasa-DFBps chr.05 5 NC_027304.1:55.566.855-55.567.573 No pred.
AGLPVIHTWETPAGGYQFQGIVDCEYDTIDNMIYFVKNIFNQKLTIYGARVQKYMGFGEY
GIRNADHYNSQAWKMAIRKA*VETICPYSARFFKLSTLERIVPPTVKVCLTKTSRYGELS
MLEDTSGLHTKWFKMAIGVCSLFIGVAMAIGGVYYWWKNR
```

AF1.2 Brown trout (*Salmo trutta;* Satr)MHCII sequences

```
>Satr-DAA1 NW_021822588.1:3.641-5.620 XP_029600114.1
MKTSVIVLVLCWQVYAEHKVLHKDLYISGCSDSDGLDMYGLDGEELWYADFIKGEGVVAL
PPFVDPLSFPGFYEQAVGQQGVCKANLATSIKAYKNPEEKIAPPHSSIYPRDDVDLGVEN
TLICHVSGFHPAPVRVRWTRNNQNLTEGVRLSTPYPNADFTLNQFSSLPFTPEEGDIYGC
TVEHKGLAEPLTRIWEPEVTQPSVGPAVFCGVGLTLGLLGVATGTFFLIKGNQCN
>Satr-DAB1 NW_021822588:9.176-13.055 XP_029600113.1
MMRQCRYSSKDLQGIELITSYVFNKVEDIRFNSTVGKYVGYTEHGVYNAEAWNSDAGILA
QELGELERFCKHNADLHYSAILDKTVAPHVRLSSVAPPTGRHPAMLMCSAYDFYPKPIRV
TWLRDGGEVKSDVTSTEELANGDWYYQIHSHLEYTPRSGEKISCMVEHISLTEPMVYDWD
PSLPEAERNKIAIGASGLVLGAILALAGLIYYKKKSSGVL
>Satr-DAA2 chr.36 NC_042992.1:504.310-507.170 XP_029591961.1
MSLCCLNMKLSVIVLILCCQVYAEDKVLHIDLYVSGCSDSDGVGVFGLDGEEKWYADFNK
GKGVVVQPPFSDPLNYDRFYEHAVGSQEILKANLAKCIKAYKNPPEKIVPPHSSIYPRDD
VELGVENTLICHVSGFFPPPVRVRWTRNNQNVTEGVRLSTPYPNADVTFNQFSSLPFTPE
EGDIYSCTVEHKGLTETLTRIWEPEVSQPSVGPAVFCGVGLTLGLLGVATGTFFLIKGNQ
CN
>Satr-DAB2 chr.36 NC_042992.1:498.192-502.800 XP_029591960.1
MSIPIAFYICLTLFLSIFYGIDGYFYHRVSECRYSSKDLQGIEYIDSFYFNQAEHVRFNS
TVGKYVGYTEYGVKSADAWNKDYLVQELGALESYCKYNAAIYYGAILDKTVAPHVRLSSV
TPPSGRHPAMLMCSAYDFYPKPIRVTWLRDGREVKSDVISTEELANGDWYYQIHSHLEYT
PRSGEKISCMVEHISLSEPMMYHCDPSLPEAERNKIAIGASGLVLGAILALAGLIYYKKK
SAGEVISV
>Satr-DBA chr.15 NC_042971.1:21.333.634-21.340.327 XP_029546517.1
MSLEMNYSVIILILTGAVCTSAEIHHEIHFIYGCFESSDPAVGLEIDGDEVFYGDFNKNS
NTCLIADVAFTLPKFISITPEDKERACEYATISRVWCKDCIAWGKQSEPKIPKIKDAPES
TIYPRDEVELGVENTLICFVNDFFPPPVKVNWTKNGMEVTEGLSLSRYYPNKDGTFHQFS
SLSFTPQKEDVYICAVAHTALKDPKTREYKVSGSSAGPGPAVFCGVGLTLGLLGVATGIF
LIYKGKRATESQEYSWEVRHWTREI
>Satr-DBB chr.15 NC_042971.1:21.322.740-21.326.263 XP_029546518.1
MYVLNFFSIHLLLLFSSLSEVVDSSDEDFAHDDAWCRFSSRDLHNMEYILEHHFNKIMVA
QYNSTTERWTGYTAWGVISAEKWNEDPDEIPRRRTDMDVLCKPYANRIYNATEMFMVEPN
VTLRLEGPSSDSSLVCSVHFFYPKHIRVTWLRNGEEVTSDVTSTDVLANGLWSYQIQSYL
KYTPTTGERITCMVEHISQTEPKLYYWDPSLPKSEKNKIVIGVCGLLLGVVFVVAGLIYW
TKSTGRLLGLIGERDYGTCD
>Satr-DCA1 chr.3 No pred. NC_042959.1:66.026.793-66.028.716
MNLSVAIVVLTAVVCTSAEVPHETVYVLGCLEKTKVEAEAELQLDGEEVVYAEFQSGQEV
WTLPEFLGPFSSSTVRNFKNAVKGRRLCRDALALWIFEEKSPPEVKDAPESTIYPRAEEE
LGVENTLICFANHFYPPPVKVNWTKNGLEVTEGTSLSRYYPNEDGTFHQFSSLSFTPQEG
DVYGCTVEHTALEDPKTRFWEYEVREVSGSSAGPAVFCGVGLTLGLLGVAIGTFLYVKGQ
QFN
>Satr-DCA2 chr.37 NC_042993.1:6.816.058-6.815.141
MNLSVTIVVLTAVVCTSAEVPHETVYVLGCLEKTKVEAELQLDGEEVVYADFQSGQEVWT
LPEFLGPFPSSSTRKLFYKNAVKGRLLCRDALALWILEENCPPEVKDAPESTIYPRAEEE
LGVENTLICFANHFYPPPVKVNWTKNGLEVTEGASLSRYYPNEDGTFHQFSSLSFTPQEG
DVYGCTVEHTALEDPKTRFW
```

```
>Satr-DCB1 chr.03 NC_042959.1:66.032.511-66.033.418 corr. XP_029601215.1
DGYFGHFEMRCWFSSEDPRDIEYLLQVYGNKKLMGQYNSTTEKCTVYTQWMKNFTETACK
GRAFLADRREEMKKYCSSNVPVVYGYLLDKAVEPYIRLRPVEPFSTRHPAMLVCSAYDFY
PKPIRVTWLRDGQEVTSNVTSTEELVNGDWTYQIHSHLEYTPTPGERIPCMVEHFSLTEP
KLHDWDPSLPGPERNKMVIGACGLLLGVVFIAAGLIYYRKKSTEGQVLVPTMALPESYGT
I
>Satr-DCB2 chr.37 NC_042993.1:6.809.351-6.814.933 XP_029594157.1
MSVLNLSSIHLLLLFSSLSGVDGYFGHFEMRCRFSSEDPQDIEYLLQVYGNKKLLGQYNS
TTEKCTVYTQWMKNFTETACKGPAFLAERREEMKKYCSSNVPVVYGYLLDKAVEPYIRLR
PVEPFSTRHPAMLVCSAYDFYPKPIRVTWLRDGQEVTSNVTSTEELVNGDWTYQIHSHLE
YTPTPGERITCMVEHFSLTEPKLYDWDPSLPGPERNKMVIGACGLLLGVVFIAAGLIYYR
KKSTEGQVLVPTMALPESYGTI
>Satr-DDA chr.14 NC_042970.1:79.981.941-79.984.210 XP_029545631.1
MMMALVFMILLFMVSSTQGQEQHVFNVINARSETEEFNMTIVVDDNEYLHVYLNNKEGVV
TLPEWVNYVDCPICVRVAESRRADLNNDIKLFSLETPEAKVPPEIKLYAKDEVKLGINNS
LVCFVNNFFPPPVQVKWTKNDENIPKGVKVGQYATNSDYTFYRFSTLTFEPQEGDIYTCI
VDHTALDEPLTRTWEFEVPPRASVGPAVFCGLGLTLGLLGVATGTFFLVKGTQCQ
>Satr-DDB chr.14 NC_042970.1:79.922.139-79.957.834 XP_029545630.1
MMACLQSYLLFVVMFSFFTPTDGHFITTLDLCYYRGEDPHDVEYIWRAISDMVKVMEYNS
TLNRFTGYTPTGKHKAEQFNADPVVLASFQTPLHFFCKYYGALAYKAGLNSNVEPSVHLR
SMTPHSDRHPSMLTCSAYKFYPKQIRVTWLRNGQEVTSNMTSSEELANGDWHYQIHSYLE
YTPTPGEKISCMVEHASFTEPKILHWDMSLPESGRNKIAIGASGLVLGVVFAAAGLLYYN
RRKTTGGGGRELVPTSHPSQ
>Satr-DEA chr.37 NC_042993.1:23.645.472-23.647.788 No pred.
MGCRVFLAFILGVCLLSHCQSKHLLRFLTFCQKNVPSDEEYDVEFGGDELFYVDPITYQV
ERRLSEFAQQWTPDPGLPHEVYVSLGTCQYNNPRCIVGEKSPPEAIEVPTSHIYSQREVE
LGVPNTLICRVSDFHPTPVDVTWTRNEQPVAERTIIQTQYYSNEDFSFRIFSYLSITPQD
GGEVEVHTDHQTVETAVCVGCVTLGVVGVATGVWFIKKAKRSAWA
>Satr-DEB LG37 NC_042993.1:23.647.912-23.651.646 XP_029593400.1
MALCWVYWMAGLSVIQTWATPAGGYQFQGIVDCEYDDTIDNMIYFVKNIFNQKLTTIYDS
RVQKYVGFEEFGIRNADRYNSQAWKMAIRKAEVETICRYSARFFKLSTLERIVPPIVKVR
LTKPSRYGEPSMLECSVLGFYPQEVRVSWLRDGLETTTAVTSTDTLANGDWSYQLHSYLE
FRPRRGESVSCMVEHPSLDEPLEVVWDTSGLDAKLFKMAIGVCSVFIGVAMAIGGGVYYW
WKNRSGFRRVNR
>satrDFA chr.3 NC_042959.1:52.808.926-52.810.975 No pred.
MGCRVFLALILGVCLLSHCQSKFAAVFDLLPKKHHSYKEYDVEFEDELFYVDPMTYRVER
RLPAFAQQWTPDPGLPHEVNVSLGTCQYNIPCCIVGENGAPNRGSHLPHILPERGDFHPT
SVDVTWTRNEQPVGEGTVSQIQYHSNENFSFRIFSYLSITPQEGDIYSCSVGHVSLQEPL
TRIWEVEVYMDHQTVETAVCVGGVTLGVVGVASGLWFIKKAKRSGWALRT
>Satr-DFB chr.03 NC_042959.1:52.808.461-52.807.743 no pred.
SGGYQFQGIVDCEYDTIDNMIYFVKNIFNQKLTIYGARVQKYMGFGEYAIRNADHYNSQA
WKMAIRKAEVETICPYSARFFKLSTLERIVPPTVKVCLTKTSRYGELSMLEGSDTSGLHT
KWFKMAIGVCSLFIGVAMAIGGVYYWWKNR
```

AF1.3 Rainbow trout (*Oncorhynchus mykiss;* Onmy) MHCII sequences

```
>Onmy-DAA Chr.17 NC_035093.1:49.670.799 -49.671.350, corr XP_021425091.1
MSLCCLNMKTSMIVLILCCQVYAEDKVLHTDIYITGCSDSDGVNMYGLDGEELWYADFNK
KEGVVALPPFADQMTFPGFYEQAVGELEIMKGNLAKCIKAYKNPPEAIDPPHSSIYPRDD
VELGVENTLICHVSGFFPPPVRVRWTRNNQNVTEGGRISTPYPNTDVTFNQFSSLSFTPE
EGDIYGCTVEHKGLTEPLTRIWEPEVSQPSVGPAVFCGVGLTLGLLGVATGTFFLIKGNQ
CN
>Onmy-DAB Chr.17 NC_035093.1:49.644.190-49.668.113 XP_021425090.1
MSMPIAFYICLTLILFIFSGTDGYFYHRLAQCRYSSKDLHGMEHIDSYVFNKAEYVRFNS
TVGRFVGYTEHGLKNAEAWNSDAGILGQEQAELERFCKHNADIYYSAIRDKTVEPHVRLS
SVTPPSGRHPAMLMCSAYDFYPKQIRVTWLRDGREVKSDVTSTEELANGDWYYQIHSHLE
YTPKSGEKISCMVEHISLTEPMMYHWDPSLPEAERNKIAIGASGLVLGTILALAGLIYYK
KKSSGVL
>Onmy-DBA Chr.12 NC_035088.1:42.256.089-42.261.363 XP_021479589.1
MSFEMNYSVIILILTGAVCTSAEIHHEIHFIYGCFESSDPAVGLEIDGDEVFYGDFSKNS
```

NTCLIADVVFTLPKFISITPEDKERACEYATISRVWCKDCIAWGKQSVPKIPKIKDAPES
TIYPRDEVELGVENTLICFVNDFFPPPVKVNWTKNGMEVTEGLSLSHYYPNKDGTFHQFS
SLSFTPQKEDVYICTVAHTALKYPKTREYKVSGSSVGPGPAIFCGVGLTLGLLGVATGIF
FIYKGKRATESQE
>Onmy-DBB chr.12 NC_035088.1:42.266.576-42.269.097 XP_021479591.1
MYVLNCFPIHLLLLFSSLSEVVDSSDEDFAHDDAWCRFSSRDLHDMEYILEHHFNKIMVA
QYNSTTERWTGYTAWGVISAEKWNEDPDEIPRRRSDMDVLCKPYANRIYNTTEMFMVEPN
VTLRLEGPSSDSSLVCSVHFFYPKHIRVTWLRNGEEVTSDVTSTDVLANGLWSYQIQSYL
KYTPTTGERITCMVEHISQTEPKLYYWDPSLPKSEKNKIVIGVCGLLLGVVFVVAGLIYW
KKSTGRLLGLIG
>Onmy-DCA1 Chr.2 NC_035078:1.949.498-1.950.031 No pred.
MNLSVAIVVLTAVVCTSAEVPHETVYVQGCLENTKVEAEAELQVDGEEVVYADFQSGREV
WTLPEFLGPFPSSTVRNFYKNAVKGRRLCQDALALWILEEKCPPEVK
>Onmy-DCA2 Chr.2 NC_035078:29.471.783-29.473.031 XP_021419620.1
MNLSVAIVVLTAVVCTSAEIPHETVYVQGCLENTKVEAEAELQVDGEEVVYADFQSGQEV
WTLPEFLGPFPSSTVRNFYKNAVKGRRLCQDALALWILEEKCPPEVKDAPESTIYPRAEE
VLGVENTLICFANHFYPPPVKVNWTKNGLEVTEGASLSRYYPNKDGTFHQFSSLSFTPRE
GDVYACTVEHTALEDPKTRFWEYKIHEVSGSSAGPAVFCGVGLTLGLLGVATGTFLYVKG
QQFN
>Onmy-DCA3ps NW_018587944.1:529-1.062 XP_021450880.1
ICFANHFYPPPVKVNWTKNGLEVTEGASLSRYYPNKDGTFHQFSSLSFTPQEGDVYACTV
EHTALEDPKTRFWEYKIHEVSGSSAGPAVFCGVGLTLGLLGVATGTFLYVKGQQFN
>Onmy-DCB1ps chr.2 NC_035078.1: 1.939.969-1.940.217 No pred.
DGYFGHFEMRCRFSSEEPRDIEFLLQVYGNKKLLGQYNSTTEKCTVYTQWMKNFTETACK
GPAFLAARRDEMKKYCS
>Onmy-DCB2 chr.2 NC_035078.1:1.942.531-1.943.589 XP_021476352.1
MSVLNLSSIHLLLLFSSLSGVDGYFGHFEMRCRFSSEEPRDIEFLLQVYGNKKLLGQYNS
TTEKCTVYTQWMKNFTETACKGPAFLAARRDEMKKYCSSNVPVVYGYLLDKAVEPYVRLR
SVEPFSTRHLAMLVCSAYDFYPKPIRVTWLRDGQEVTSNVTSTEELVNGDWTYQIHSHLE
YTPTPGERIACMVEHFSLTEPKLYDWDPSMPGPEKNKMVIGACGLLLGVVFIAAGLIYYR
KKSTEGRVLVPTMALPEIYGTI
>Onmy-DCB3ps chr.2 NC_035078.1: 1.944.223-1.944.501 No pred.
DGYFGHFEMRCRFSSEEPRDIEFLLQVYGNKKLLGQYNSTTEKCTVYTQWMKNFTETACK
GPAFLAARRDEMKKYCSSNVPVVYGYLLDKAGE
>Onmy-DCB4 chr.2 NC_035078.1: 29.466.615-29.469.923 XP_021419604.1
DGYFGHFEMRCRFSSEEPRDIEFLLQVYGNKKLLGQYNSTTEKCTVYTQWMKNFTETACK
GPAFLAARRDEMKKYCSSNVPVVYGYLLDKAVEPYVRLRSVEPFSTRHLAMLVCSAYDFY
PKPIRVTWLRDGQEVTSNVTSTEELVNGDWTYQIHSHLEYTPTPGERIACMVEHFSLTEP
KLYDWDPSMPGPEKNKMVIGACGLLLGVVFIAAGLIYYRKKSTEGRVLVPTMALPEIYGT
I
>OnmyDCB5ps NW_018528397.1:1299-1577 No pred.
DGYFGHFEMRCRFSSEEPRDIEFLLQVYGNKKLLGQYNSTTEKCTVYTQWMKNFTETACK
GPAFLAARRDEMKKYCSSNVPVVYGYLLDKAGE
>Onmy-DDA GBTD01042060.1 TSA: no genomic match
MMMALVFMIVLFTVSSTQGQAHVFTVIGARSETEEFNMTIVADGNEYLHVYLKNKEDVVT
LPEWINDVDCPICVRIAESRRAGLSNYIKLFSLETPEAKVPPEIKLYAKEEVKLGINNSL
VCFINNFFPPPVQVKWTKNDENVPKGVKVGQYATNSDYTFYRFSTLTFEPQEGDIYTCIV
DHMALDEPLTRTWEFEVPPHPSVGPAVFCGLGLTLGLLGVATGIFFLVKGTQRQ
>Onmy-DDB1 chr.13 NC_035089.1: 5.368.697-5.370.479, XP_021412280.1 corr.
MMACLQSYLLFVVMFSFFTPTDGHFITILDLCYYRGEDPHDVEYIWRAISDMVKVMEYNS
SLNRLTGYTPIGIHKAEQFNADPVVLASFQTPLNFLCKRYGAVAYEAGVNNNVEPSVHLK
SMTAHGDRHPSMLTCSAYKFYPKQIRVTWLRNGQEVTSNMTSSEELANGDWHYQIHSYLQ
YTPTPGEKISCMVEHASFTEPKILHWDMSLPESERSKIAIGPSGLVLGVVFAAAGLLYYN
RRKTTGGDVSISHDGAAVTLRPTI
>Onmy-DDB2 Chr.17 NC_035093.1: 4.571.316-4.572.534 No pred.
DMSLPESERSKIAIGPSGLVLGVVFAAAGLLYYNRRKTTGGDVSISHDGAAVTLRPTI
>Onmy-DEAps Chr.3 NC_035079.1: 28.508.845-28.509.058 No pred.
KHLLRFLTFCQKNVTSDEEYDVEFDGDELFYPAVDPMTWSLMEMSSSTQQWTPDPGLPHD
VYVSLGTCQCNVTRCIVGEKSPPEAIGG
>Onmy-DEBps Chr.3 NC_035079.1:28.510.176-28.511.183, XP_021449969.1
MALCWVYWVAGLSAIQTRATPAGGG*QFQGILDCEYDDTTDNMIYFVKNIFNQKLTTIYD

SRVQKYVGFGEFGIRNADHYNSQAWEMAIRKAEVETICRYSARYFKLSTLERIVPPIVKA
RLTKPSRYAELSMLECSVLGFYPQEVRVSWLRDRLETTIAVTSTDTLANGDWSYQLHSYL
EFRPQRGESVSCMVEHPSLDEPLEVVW
>Onmy-DFAps Chr 2 NC_035078.1: 18.600.094-18.601.625 No pred.
NHLLQFLTFCQKNITSDKEYEVEFDGDELFYVDPMTYRVEQ*LSEFAQ*WTPDPGLPH*V
YVSLGTCKYNIPHCIVGENEHYPHSGVNNRASHI*SQRKVELGVPNTLICLVINFHPTPV
HVTWTINEQPVGERTVSQTQYYSNEDFSFRIFSYLSITPQEGDIYSCSVGHVSLQEPFTR
NWEVEVHTAHQTVETAVCVGSVTLGVVGVATGVWFIKKAKRSGWA

AF1.4 Sockeye salmon (*Oncorhynchus nerka;* Onne)MHCII sequences

>Onne-DAA NW_021807294.1:41.778-44.505 XP_029508585.1
MSLCCLNMKTSVIVLILCCQVYAEDKVLHIDIYLSGCSDSDGVDMYGLDGEEEWYADFNK
KEGVVALPPFADQITFPGFYEQAVGNQGVCKANLATSIKAYKNPPETIDPPHSSIYPRDD
VELGVENTLICHVSGFFPPPVRVRWTRNNQNVTEGGRISTPYPNTDVTFNQFSSLSFTPE
EGDIYGCTVEHKALTEPLTRIWEPEVSQPSVGPAVFCGVGLTLGLLGVATGTFFLIKGNQ
CN
>Onne-DAB NW_021807294.1:46.283-52.355 XP_029508586.1
MSMPIAFYICLTLILFIFSGTDGYFYHRLAQCRFSSKDLHGIELIDSYVFNKAEYVRFNS
TVGRYVGYTEYGVKNAKAWNSDAGILGQEQAELERVCKTGAAIDYSNILDKTVEPHVRLS
SVTPPSGRHPAMLMCSAYDFYPKPIRVTWLRDGREVKSDVTSTEELANGDWYYQIHSHLE
YTPKSGEKISCMVEHISLTEPMMYHWDPSLPEAERNKIAIGALVWCWEPS
>Onne-DBA chr.22 NC_042556.1:38.336.566-38.342.193 XP_029483109.1
MSFEMNYSEIVLILTGAICTSAEIHHEIHFIYGCFESSDPAVGLEIDGDEVFYGDFNKNS
NTCLIADVVFTLPKSISITPEDKERACEYATISRVWCKNCIAWGKQSEPKIPKIKDAPES
TIYPRDEVELGVENTLICFVNDFFPPPVKVNWTKNGMEVTEGLSLSRYYPNKDGTFHQFS
SLSFTPQKEDVYICAVAHTALKDPKTREYKVSGSSVGPGPAVFCGVGLTLGLLGVATGMF
FIYKGKRAT
>Onne-DBB chr.22 NC_042556.1:38.326.373-38.330.559 XP_029483107.1
MYVLNCLSIHLLLLFSSLSEVVDSSDEDFAHDDAWCRFSSRDLHNMEYILEHHFNKIMVA
QYNSTTERWTGYTAWGVISAEKWNEDPDEIPRRRSDMDVLCKPYANRIYNTTEMFMVEPN
VTLRLEGTSSDSSLVCSVHFFYPKHIRVTWLRNGEEVTSDVTSTDVLANGLWSYQIQSYL
KYTPTTGERITCMVEHISQTEPKLHYWDPSLPKSEKNKIVIGVCGLLLGVVFVVAGLIYW
KKSTGRLLDLIGELDYGTCD
>Onne-DCA1 NW_021791902.1:24-1.239 XP_029503552.1
MNLSVAIVLLTAVVCTSAEIPHETVYVQGCLEKTKVEAEAELQVDGEEVVYADFQSGQEV
WTLPEFLGPFPSSTVRNFYKNAVKGRRLCQDALALWILEEKFPPEVKDAPESTIYPRAEE
VLGVENTLICFANHFYPPPVKVHWTKNGLEVTEGASLSRYYPNKDGTFHQFSSLSFTPQE
GDVYACTVEHTALEDPKTRFWGEKLFL
>Onne-DCA2ps NW_021792314:1-276 XP_029503714.1
IPPPDAPESTIYPRAEEVLGVENTLICFANHFYPPPVKVHWTKNGLEVTEGASLSRYYPN
KDGTFHQFSSLSFTPQEGDVYACTVEHTALEDPKTRFWEIHEVSGSSAGPAVFCGVGLTL
GLLGVATGTFLYVKGQQFN
>Onne-DCBps NW_021784836.1:4-1.153 XP_029500393.1
LDKAVEPYIRLRSVEPFSTRHLAMLVCSAYDFYPKPIRVTWLRDGQEVTSNVTSTEELVN
GDWTYQIHSHLEYTPTPGERIACMVEHFSLTEPKLYDWDPSMPGPEKNKMVIGACGLLLG
VVFIAAGLIYYRKKSTEGRVLVPTMALPESYGTI
>Onne-DDB NW_021797582.1:351-1498 corr XP_029505744.1
FMVHSCFFSPDGHFITILDLCYSRGEDPHVEYIWRAISDLVKVMEYNSSLNRLTGYTPIG
IHKAEQCNADPVVLASFQTPLLSFCKHYGAVAYEAGVNNNHPSMLMCSAYKFYPKQIRVT
WLRNGQEVTSHMTSSEELANGDWHYQIHSYLEYTPTPGEKISCMVEHASFTEPKILHWIP
DMSLPESERSKIGLVLGVVFAAAGLYYNRRKTTGGDGE
>Onne-DEB chr.03 NC_042537.1:11.058.502-11.059.845 corr. XP_029481671.1
MALCWVYWVAGLSAIQTRATPAGGYQFQGIVDCEYDDTTDNMIYFVKNIFNQKLTTIYDS
RVQKYVGFGEFGICNADHYNSQAWKMAIRKAEVETICRYSARFFKLSTLERIVPPIVKAR
LTKPSRYGELSMLECSVLGFYPQEVRVSWLRDWLETTTAVISTDTLANGEWSYQLDSYLE
FRPQRGERVSCMVEHPSLDEPLEVVDTSGLDAKWFKMAIGLCSLFNGVAMAIGGGVYYWW
KNR
>Onne-DEA chr.03 NC_042537:11.054.824-11.056.805 No pred.
MGCRVFLAFIRGVCLLSHCQSKHLLRFLTFCQKNGTSDEEYDVEFDGDELFYGLPHDVYV

SLGTCQYNIPRCIVGEKSPPEAIEVPTSHIYFQREVELGVPNTLICQVSDFHSTPVDVTW
TRNEQPVGERTVIQTQYYSNKDISFRIFSYLSITPQEGDIYSCSVGNVSLQEPLTRIW
>Onne-DFAps Chr.14 NC_042548.1:12.356.454-12.358.337 No pred.
MGCRVFLALILGVCLLSHRQSKHFLRFLTFCQNNITSDKEYDVEFDGDELFYVDPMIYWV
EQRLSEFAQ*WTPDPGLPH*VYVSLGTCQYNIPHCIVGEHYPHSGVNNRASHI*SQRKVE
LGVPNTLICQVINFHPTPVDVTWTINEQPVGERTVSHTQYYSNEDFSFRIFSYLSITPQE
GDIYSCSVGHISLQEPFTRNWEVEVHTTHQTVERAVCVGSVTLGVVGVATGVWFIKKAKR
SGWALRT

AF1.5 Coho salmon (*Oncorhynchus kisutch*; Onki)MHCII sequences

>Onki-DAA chr.01 NC_034174.2:30.733.108-30.738.210 XP_031673251.1
MSLCCLNMKTSMIVLILCCQVYAEDKVLHIDIYITGCSDSDGVDMYGLDGEELWYADFNK
KEGVVALPPFADQISFPGFYELAVGNQGVCKANLATFIKAYKNPPETIDPPHSSIYPRDD
VELGVENTLICHVSGFHPAPVRVRWTRNNQNVTEGVRISTPYPNTDFTLNQFSSLSFTPE
EGDIYGCTVEHKALTEPLTRIWEPEVSQPSVGPAVFCGVGLTLGLLGVATGTFFLIKGNQ
CN
>Onki-DAB chr.01 NC_034174.2:30.740.885-30.746.625 XP_031673186.1
MSMPIAFYICLTLLWSIFSGTDGYFHQVVSQCRYSSKDLHGIEFIDSYVFNRVEHIRFNS
TVGRYVGYTELGLKNAEAWNKGPQLGQEQGELERFCKRNADLHYRAILDKTVEPHVRLSS
VTPPSGRHPAMLMCSAYDFYPKQIRVTWLRDGREVKSDVTSTEELANGDWYYQIHSHLEY
TPKSGEKISCMVEHISLTEPMMYHWDPSLPEAERNKIAIGASGLVLGTILALAGLIYYKK
KSSGVL
>Onki-DBA chr.06 NC_034179.2:46.329.716-46.335.674 XP_020340180.1
MSFEMNYSVIILILTGAACTSAETHHEIHFIFGCFESSDPAVGLEIDGDEVFYGDFNKNS
NTCLIADVVFTLPKFVSITPEDKERACEYATISRVWCKDGIAWGKKSEPKIPKIKDAPES
TIYPRDEVELGVENTLICFVNDFFPPPVKVNWTKNGMEVTEGLSLSRYYPNKDGTFHQFS
SLSFTPQKEDVYICAVVHTALKDPKTREYKVSGSSVGPGPAVFCGVGLTLGLLGVATGIS
FIYKGKRATESQE
>Onki-DBB chr.06 NC_034179.2:46.340.511-46.343.391 XP_020339901.2
LQIRLLKNTFTCPFSVIIVDSSDEDFAHNDAWCRFSSSDLHDMEYILEHHFNKIMVAQYN
STTERWTGYTAWGVISAEKWNEDPDEIPRRRSDMDVLCKPYANRIYNTTEMFMVEPNVTL
RLEGPSSDSSLVCSVHFFYPKHIRVTWLRNGEEVTSDVTSTDVLANGLXSYQIQSYLKYT
PTTGERITCMVEHISQTEPKLYYWDPSLPKSEKNKIVIGVCGLLLGVVFVVAGLIYWKKS
TGRLLDLIGELDYGTCD
>Onki-DCA1 NW_022263943.1:7.643-10.265 XP_031675123.1
MNLSVAIVVLTAVVCTSAEIPHETVYVQGCLEKTKVEAEAELQVDGEEVVYADFQSGQEV
WTLPEFLGPFPSSTVHNFYKNAVKGRRLCQDALALWILEEKFPPEVKDAPESTIYPRAEE
VLGLENTLICFANHFYPPPVKVNWTKNGLEVTEGASLSRYYPNKDGTFHQFSSLSFTPQE
GDVYACTVEHTALEDPKTRFWEIHEVSGSSAGPAVFCGVGLTLGLLGVATGTFLYVKGQQ
FN
>Onki-DCB1 NW_022263943.1:2.007-7.643 XP_031675120.1
MSVLNLSSIHLLLLFSSLSGVDGYFGHFEMRCRFSSEEPRDIEFLLQVYGNKKLLGQYNS
TTEKCTVYTQWMKNFTETACKGPAFLAARREEMKKYCSSNVPVVFGYLLDKAVEPYIRLR
SVEPFSTRHLAMLVCSAYDFYPKPIRVTWLRDGQEVTSNVTSTEELVNGDWTYQIHSHLE
YTPTPGERIACMVEHFSLTEPKLYDWDPSMPGPEKNKMVIGACGLLLGVVFIAAGLIYYR
KKSTEGRVLVPTMALPESYGTI
>Onki-DCA2 NW_022261979.1:311.232-314.542 XP_031667867.1
MNLSVAIVVLTAVVCTSAEIPHETVYVQGCLEKTKVEAEAELQVDGEEVVYADFQSGQEV
WTLPEFLGPFPSSTVHNFYKNAVKGRRLCQDALALWILEEKFPPEVKDAPESTIYPRAEE
VLGLENTLICFANHFYPPPVKVNWTKNGLEVTEGASLSRYYPNKDGTFHQFSSLSFTPQE
GDVYACTVEHTALEDPKTRFWEIHEVSGSSAGPAVFCGVGLTLGLLGVATGTFLYVKGQQ
FN
>Onki-DCB2 NW_022261979.1:314.625-319.616 XP_031667866.1
MSVLNLSSIHLLLLVSSLSGVDGYFGHFEMRCRFSSEEPRDIEFLLQVYGNKKLLGQYNS
TTEKCTVYTQWMKNFTETACKGPAFLAARREEMKKYCSSNVPVVFGYLLDKAVEPYIRLR
SVEPFSTRHLAMLVCSAYDFYPKPIRVTWLRDGQEVTSNVTSTEELVNGDWTYQIHSHLE
YTPTPGERIACMVEHFSLTEPKLYDWDPSMPGPEKNKMVIGACGLLLGVVFIAAGLIYYR
KKSTEGRVLVPTMALPESYGTI
>Onki-DDB1 NW_022265017.1:30.916-38.225 XP_031676050.1
MDGSHPGGPQLLQSNYTDQQCHGDDPDGHFITILDLCYSRGEDPHDVEDIWRAISDLVKV

MEYNSSLNRFTGYTPIGIHTAEQLNADPVVLASFQTPLHFFCKHYGAVAYEAGLNNNVEP
SVHLSMTPHGDRHPSMLTCSAYKFYPKQIRVTWLRNGQEVPSNMTSSEELANGDWHYQIH
SYLEYTPTPGEKISCMVEHTSFTEPKILHWDMSLPESERSKIAIGASGLVLGVVFAVAGL
LYYNRRKTTGGDGEYKMFYCQIHRIHGLMMVSILEMCKECLLLF
>Onki-DDB2 NW_022261978.1:2.892.149-2.893.573 XP_031666909.1
VHSCFFSPDGHFITILDLCYYRGEDPHDVEDIWRAISDMVKVMEYNSPVVLASFQTPLHF
FCKHYGAVAYEAGLNNNHLSMTPHGDRHPSMLTCSAYKFYPKQIRVTWLRNGQEVPSNMT
SSEELANGDWHYQIHSYLEYTPTPGEKISCMVEHTSFTEPKILHWDMSLPESERSKIAIG
ASGLVLGVVFAVAGLLYYNRRKTTGGDGE
>Onki-DEA 1 chr.02 NC_034175.2:19.663.235-19.668.779 XP_031657800.
MGCRVFLAFILSVCLLSHCQTLLPSRGQLTQGLPHDVYVSLGTCQYNIPRCIVGEKSPPE
AIEVPTSHIYSQREVELGVPNTLICQVSDFHPTPVDVTWTRNKQPVGERTVIQTQYLSNK
DFSFRNFSYLSITPQEGDIYSCSVGHVSLQEPLTRIWEVEVDTDHQTVETAVCVGGVTLG
VVGVASPGRSGATSSTPT
>Onki-DFAps chr.13 NC_034186.2:59.583.236- 59.585.022 No pred.
MGGRVFLALILGVCLLSHHQSGNHLLRFLTFCQNNITSDKEYDVEFDGDELFYVDPMTYW
VDQRLSEFAQ*WTPDPGLPRVYVSLGTCQYNIPHCIVGEHYPHSGVNNRASHI*SQRKVD
LGVPNTLICQVINFHSTPVDVTWTINEQPVGERTVSQTQYYSNEDFSFRIFSYLSITPQE
GGDIYSCSVGHISLQEPFTRNWEVEVHTAHQTVETAVRVGSVTLEVVGVATGVWFIKKAK
RSGWALRT

AF1.6 Chinook salmon (*Oncorhynchus tshawytscha*)MHCII sequences

>Onts_DAA NW_020130363.1:60.941-65.768 XP_024264231.1
MSLCCLNMKTSMIVLILCCQVYAEDKVLHIDISINGCSDSDGVGMYGLDGEELWYADFNK
KEGVMPLPPFADQMTFPGFYEQAVGNQGVCKANLATSIKAYKNPPETIDPPHSSIYPRDD
VKLGVENTLICHVSGFFPPPVRVRWTRNNQNVTEGGRISTPYPNTDVTFNQFSSLSFTPE
EGDIYGCTVEHKGLTEPLTRIWEPEVSQPSVGPAVFCGVGLTLGLMGVATGTFFLIKGNQ
CN
>Onts-DAB NW_020130363.1:54.045-59.238 XP_024264230.1
MSMPIAFYICLTLILFIFSGTDGYFHHRLVQCRYSSKDLHGIEFIDSYVFNKAEYIRFNS
TVGRYVGYTELGVKNAEAWNKGPQLGQEQAELERFCKPNAALHYRAILDKTVEPHVRLSS
VTPPSGRHPAMLMCSAYDFYPKQIRVTWLRDGREVKSDVTSTEELANGDWYYQIHSHLEY
TPKSGEKISCMVEHISLTEPMMYHWDPSLXELNRNKIAIGAXGLWLGTILALAGLIYYRK
KSSGVL
>Onts-DBA1 chr.09 NC_037105.1:43.135.612-43.141.617 XP_024287887.1
MSFEMNYSVIIILILTGAVYTSAEIHHEIHFIYGCFESSDPAVGLEIDGDEVFYGDFNKNS
NTCLIADVVFTLPKFISITPEDKERACEYATISRVWCKDCIAWGKQSKPKIPKIKDAPES
TIYPRDEVELGVENTLICFVNDFFPPPVKVNWTKNGMEVTEGLSLSRYYPNKDGTFHQFS
SLSFTPQKEDVYICAVAHTALKDPKTREYKVSRSSVGPGPAVFCGVGLTLGLLGVATGIF
FIYKGKRATESQE
>Onts-DBB1 chr.09 NC_037105.1:43.147.513-43.149.018 No pred.
AHDDAWCRFSSRDLHDMEYILEHHFNKIMVAQYNSTTERWTGYTAWGVISAEKWNEDPDE
IPRRRSDMDVLCKPYANRIYNTTEMFMVEPNVTLRLEGPSSDSSLVCSVHFFYPKHIRVT
WLRNGEVTSDVTSTDVLANGLWSYQIQSYLKYTPTTGERITCMVEHISQTEPKLYYWDPS
LSKSEKNKIVIGVCGLLLGVVFVVAGLIYWKKSTGEE
>Onts-DBA2 chr.09 NC_037105.1:55.361.845-55.367.850 XP_024288155.1
MSFEMNYSVIIILILTGAVYTSAEIHHEIHFIYGCFESSDPAVGLEIDGDEVFYGDFNKNS
NTCLIADVVFTLPKFISITPEDKERACEYATISRVWCKDCIAWGKQSKPKIPKIKDAPES
TIYPRDEVELGVENTLICFVNDFFPPPVKVNWTKNGMEVTEGLSLSRYYPNKDGTFHQFS
SLSFTPQKEDVYICAVAHTALKDPKTREYKVSRSSVGPGPAVFCGVGLTLGLLGVATGIF
FIYKGKRATESQE
>Onts-DBB2 chr.09 nNC_037105.1:55.372.711-55.376.082 XP_024288156.1
MYVLNCFSIHLLLLFSSLSEVVDSSDEDFAHDDAWCRFSSRDLHDMEYILEHHFNKIMVA
QYNSTTERWTGYTAWGVISAEKWNEDPDEIPRRRSDMDVLCKPYANRIYNTTEMFMVEPN
VTLRLEGPSSDSSLVCSVHFFYPKHIRVTWLRNGEEVTSDVTSTDVLANGLWSYQIQSYL
KYTPTTGERITCMVEHISQTEPKLYYWDPSLSKSEKNKIVIGVCGLLLGVVFVVAGLIYW
KKSTGRLLGLIGELDYGTCD
>Onts-DCA1 chr.23 NC_037119.1:991.276-992.667 XP_024241033.1
YADFQSGQEVWTLPEFLGPFPSSTVRNFYKNAVKGRRLCQDALALWILEEKFPPEVKDAP
ESTIYPRAEEVLGVENTLICFAIXFYPPPVNVNWTKNGLEVTEGVSLSRYYPNKDGTFHQ

FSSLSFTPQEGDVXSLHVEHTALEDPKTRFWEIHGVSGSSAGPAVFCGVGLTLGLLGVAT
GTFLYVKGQQFN
>Onts-DCB chr.23 NC_037119.1:986.253-990.499 XP_024241171.1
MRCRFSSEEPRDIEFLLQVYGNKKLLGQYNSTTEKCTVYTQWMKNFTETACKGPAFLAAR
RDEMKKYCSSNVPVVYGYLLDKAVEPYIRLRSVEPFSTRHLAMLVCSAYDFYPKPIRVTW
LRDGQEVTSNVTSTEELVNGDWTYQIHSHLEYTPTPGERIACMVEHFSLTEPKLYDWDPS
MPGPEKNKMVIGACGLLLGVVFIAAGLIYYRKKCNEGRVLXPTMALPESYGTI
>Onts-DCA2 NW_020138771.1:20-1.199 XP_024267460.1
MNLSVAIVVLTAVVCTSAEIPHETVYVQGCLEKTKVEAEAELQVDGEEVVYADFQSGQEV
WTLPEFLGPFPSSTVRNFYKNAVKGRRLCQDALALWILEEKFPPEVKDAPESTIYPRAEE
VLGVENTLICFANHFYPPPVNVNWTKNGLEVTEGVSLSRYYPNKDGTFHQFSSLSFTPQE
GDVYACTVEHTALEDPKTRFWEIHEVSGSSAGPAVFCGVGLTLGLLGVATGTFLYVKGQQ
FN
>Onts-DDB chr.18 NC_037114.1:40.224.963-40.227.299 No pred.
LWFIHVFSHQMDTITILDLCYSRGEDPHDVEYIWRAIPDLVKVMEYNSSLNRLTGYTPIG
THNAEQLNTDPVVLASFQTPLHFFCKHYGAVAYEAGVNNNVEPSVHLSMRPHGDRHPSML
TXSAYNFYPKQIRVTWLRNGQEVTSNMTSSEELANGDWHHQIHSYLEYTPTPGEKISCIV
EHTSFTEPKILHWDMSLPESERSKMAIGASGLVLGVVFAAAGLLYYNRRKTTGGDGE
>Onts-DEAps chr.03 NC_037099.1:16.108.478-16.110.728 No pred.
MGCRVFLAFILGVCLLSHCQSKHLLRFLTFCQKNVTSDEEYDVEFDGDELFYGLPHDVYV
SLGTCQYNIPRCIVGEKSSPEAIEVPTSDIYSQREVELGVPNTLICQVSDFHPTPVDVTW
TRNEQPVGERTVIQTQYYSNKDFSFRIFYLSITPQEGDIYSCSVGHVSLQELPRSWEVEV
DTDH*TVETAVCVGGVTLGVVGVASGVWFIKKAKRSGWALRT
>Onts-DEB chr.03 NC_037099.1:16.111.442-16.115.521 XP_024251641.1
MHHGSLLGLLGGWTFFHSDPGNTSRAQKYVGFGEFGIRNADHYNSQAWKMAIRKAEVETI
CRYSARFFKLSTLERIVPPIVKVRLTKPSSYGELSMLECSVLGFYPQEVRVSWLRDGLET
HSXVTSTDTLANGDWSYPLHSYLEFRPQRGESVSCMVEHPSLDEPLEVVWGERPGEEGVL
WSGVRAGGRGGNVGERGTERGKNILLIAVFS
>Onts-DFAps chr.23 NC_037119.1:13.088.214-13.090.095 No pred.
MGGRVFLALILGVCLLSHRQSKHLLRFLTFCQKNITSDKEYEVEFDGDELFYVDPMTYRV
EQ*LSEFAQ*WTPDPGLPH*VYVSLRTCQYNIPHCIVAEHYPHSGVNNRASHI*SQRKVE
L*VPNTLICQVINFHSTPVDVTWTINEQPVGERTISQTQYYSNEDFSFRIFSYLSITPQE
GDIYSCSVGHISLQEPFTRNWEVEVHTAHQTVETAVCVGSVTLGVVGVATGVWFIKKAKR
SGWALRT

AF1.7 Charr (*Salvelinus alpinus/malma*)MHCII sequences

>Saal-DAA NW_019945608:1634-3556 corr. XP_023999496.1
MSLCCLNMKTSVIVLILCCQVYAEDKLLHIDLYIAGCSDSDGVDMYGLDGEELLYADFKK
GEVVMPLPPFADPFTYPGAYEQAVGNQGACKANLATSIKAYKNPPETIDPPHSSIYPRDD
VELGVENTLICHVSGFHPAPVRVRWTRNNQNVTEGVRTSTPYPNTDFTLNQFSSLTFTPE
EEDIYGCTVEHKGLTEPLTRIWEPAPVWSAVFCGVGLTLGLLGVATGTFFLIKGN
>Saal-DAB NW_019945608:6748-11843 XP_023999497.1
YSSKDLHGAEFIDSYVFNKAEYVRFNSTVGKYVGYTEHGVYNAKTWNKGSELVRELGELE
RFCKHNADIHYSAILDKTVEPHVRLSSVTPSSGRHPAMLMCSAYDFYPKPIRVTWLRDGH
EVKSDVTSTEELANGDWYYQIHSHLEYTPKSGEKISCMVEHISLTEPMMYHWDPSLPEAE
RNKIAIGASGLVLGAVLALAGLIYYKKKSSGVL
>Saal-DBA chr.20 NC_036860.1: 30.150.199-30.155.979 XP_023866627.1
MSFEMNYSVIIILILTGAVCTSAEIHHEIHFIYGCFESSDPAVGLEIDGDEVJYGDFNKNS
NTCLIADVVFTLPKFISITPEDKEXACEYATISRVWCKNCVAWGKQSEPKIPKIKDAPES
TIYPRDEVELGWENTLICFVNDFFPPPVKVNWTKNGMEVTEGLSLSRYYPNKDGTFHQFS
RLSFTPQKEDGYICAVVHTALKDPKTREYKVSGSSAGPGPAVFCRVGLTLGLLGVATGIF
FIYKGKRATESQE
>Saal-DBB chr.20 NC_036860.1:30.142.406-30.145.022 XP_023868529.1
MYVLNCFSIHLLLLFSFLSEVVHSSDEDFAYNDVWCRFSSRDLHDMEYILEHHFNKIMVA
QYNSTTERWTGYTALGVISAEKWNEDPDEIPRRRTDMDVLCKPYATRIYNTVEMFMVEPN
VTLRLEGPSSDSSLVCSVHFFYPKHIRVTWLRNGEEVTSDVTSTDILANGLWSYQIQSYL
KYTPTTGERIICMVEHISQTEPKLYHWDPSLPKSEKNKIVIGVCGLLLGVVFVVAGLIYW
KKSTGRLSGLIGECDYGTCD
>Saal-DCAps NW_019944863.1:7.697-8.641 No pred.

MNFCVAIVVLTAVVCTSAESHETVYVLGCLEKTKVEAEAALQVDGEK*YMLTSRVTGSLD
IA*VLGPFQALLSVTSYKNAVKGRRLCRDALALWILEEKGPPEVKVENTLICFANHFYPP
PVKVNWTKNGLEVTEGASLSRYYPNEDGTFHQLSSLSFTPQEGDVYACTVEHTALEDPKT
RFW
>Saal-DCB1ps? NW_019944863.1:12.805-13.947 pseudogene
MKNFTETACKGPAFLAARREEMKKYCSSNVPVVYGYLLDKAVEPYIRLRSVEPFSTRHLA
MLVCSAYDFYPKPIRVTWLRDGQEVTSNVTSTEELVNGDWTYQIHSHLEYTPTLERESPV
WWSTSASLSPNCMTGTPPCLVLRGIRW
>Saal-DCB2 NW_019947682:21.354-23.308 XP_024000748.1
DGYFGRFEMRCRFSSEDPRDIEFLLQVYGNKKLLGQYNSTTEKCTVYTQWMKNFTETACKG
PAFLAARREEMKKYCSSNVPVVYGYLLDKAVEPYIRLRSVEPFSTRHLAMLVCSAYDFYP
KPIRVTWLRDGQEVTSNVTSTEELVNGDWTYQIHSHLEYTPTPGERITCMVEHFSLTEPK
LYDWDPSMPGPERNKMVIGACGLLLGVVFIAAGLIYYRKKSTEGRVLVPTMALLESYGTI
>Saal-DDAps NW_019945231:17.363-22.556 XP_023999181.1 corr.
HVFNVINARSETEEFNMTVVXDGNEYLHVYLNNKEDVVTRPEWGNYIDCPICVRFAERQR
AGLNNSIKVLSLETPEAKVPPEIKLYAKEEVNLGINNSLXCFVNXFFPPPVQVKWTKNDE
NVPKGVKAGQYATNSDYTFYHFSTLTFEPQEGDIYTCIVDHTALDEPLTRTWEFEVPPP*
GPAVFCGLGLTLGLLGVATGTFFLVKGTQCQ
>Saal-DDB1 NW_019945231:30.347-33.914 No pred.
FFSPDVHFVTTLELCYYRGEDPQHVSSSSESINMVTVAEYNSAFNRYTGYTVGIYHAEIQ
NKDLFFYCKDDGELVYKAGFNKVEPSIHLRSMTPHPSKLTCSSYKFNPKQIRVTXWRDGQ
EVTSDATSPEDLANGNGYHQIHTYLEYTPIPERISCMVKHASFTEPKSLHWSGLVLGVFF
GAVGLDYYNRRKTTCG
>Saal-DDB2ps NW_019952256:1.709-3.388 XP_024001573.1
NSTLNRFM*GYTPFGIHTRAI*EQFNADPVVL*ASFQTPLHFFCKHYGALSTKVEPSVHL
RSMTXHSDRHPSMLTCSAYKFYPKQIRVTWWRDGQEVTSNMTSSEELSNGDWHYQIHSYL
EYTPTPGEKISCMVEHASFTEPKILHWDMSLPGSERSKIAIGASGLVLGLVFAAAGLLYY
NGRKTTGGDGRELVPTSHPSQ
>Saal-DDB3 NW_019944305.1:90.396-105.291 no pred.
MVACLQSYLLFVVMFSFFTPTDGHFMTSLDLCYYRGEDPHDVEYVWRAISDMVKVMEYNS
TLNRFTGYTPFGIHIAEQFNADPVVLASFQTPLHFFCKHYGALVYEAGINNNVEPSVIWR
SMTPHSDRHPLMLTCSAYKFYPKQIRVTWWRDGQEVTSNMTSSEELSNGDWHYQIHSYLE
YTPTPGEKISCMVRHASFTEPKILHWDMSLPGSERSKIAIGASGLVLGLVFAAAGLLYYN
GRKTTGG
>Saal-DEA chr.35 NC_036874.1: 9.075.676-9.078.023 No pred.
MGCRVFLAFILGVCLLSHCQSGKHLLRFLTFCQKNIPSDEEYDVEFDGDELFYVDPITYR
VERRLSEFAQQWTPDPGLPHEVYVSLGTCQYNIPRCIVGEKSPPEAIHIYSQREVELGVP
NTLICRVSDFHPTPVDVTWTRNEQPVGERTIIQTQYYSNKDFSFRIFSYLSITPQEGDIY
SCSVGHVSLQEPLTRIWEVEVHTDHQTVETAVCVGGVTLGVVGVATGVWFIKKAKRSGWA
LRT
>Saal-DEBps chr.35 NC_036874.1:9.078.877-9.079.056 No pred.
VSPSVKVRLTKPSRYGELSMLECSVLGFYPQEVRVSWLGDGLETTTAVTSTDTLANGDWS
>Saal-DFA chr.6.1 NC_036845: 13.836.333- 13.838.339 No pred.
MGCRVFLALILGVCLLSYCQSKHLLLFLIFCQKNITSNKEYDVEFAGDELFYVDPMTYRV
EQRLSEFAQQWTPDPGLPQEVYVSLGTCQYNIPHCIVGENSHIYSQRKVEXGVPNTLICR
VINFHPTPVDVTWTRIEQPVGERTVXQTQYYSNEDFRIFSYLSITPQEGDIYSCSVGHVS
LQEPLTRIWEVEVHTDHQTVTTAVCVGSVTLGVVGVVTGVWFIKKAKRSGWALRT
>Saal-DFBps chr.6.1 NC_036845: 13.838.788-13.839.510 No pred.
SGGYQFQGIVDEYDDTIDDMIYFV*KNIFNQKLTIYGSRVQKYMGFGEYGICNADHYNSQ
AWKME*VETICPYSARFFKLSTLERIKVCLTKPSR*GELSMLEGS*VINFHPTPVDVTWT
RIEQPVGERTVXQTQYYSNEDFRIFSYLSITPQEGDIYSCSVGHVSLQEPLTRIWDTSCL
DTKWFKMAIVVCSLFIGVAVAIGGFYSWWKNR

AF1.8 Northern pike (*Esox Lucius*) MHCII sequences

>Eslu-DAB1 XP_034143025.1 chr.17 NC_047585.1:25.147.505-25.152.712
MCLFHYHSQQGNMAIQNIYIYLLGFLSISYRTDGYYEQVVDECRFTSKDLQDAEFIRSYV
FNMVEDVRFNSSVGEYVGYTELGVKNAKAWNSDPGELAGIRAQLDSYCRNNAGIHYSAVL
DKTAKPYVRMSSVTPPSGRHTAMLMCSAYDFYPKQIRVTWQRDGHEIKSDVTSTEELADG
DWYYQIHSHLEYTPKSGEKISCMVEHSSLPEPMVYDWDPSMPESERNKIAIGASGLVLGA
IIALAGLVYYKKKSAGVL

>Eslu-DAA1 NP_001290803.1 chr.17 NC_047585.1:25.160.320-25.165.305
MKSSVIFLILSSVVYAEIKVLHIDLYTDGCSDSDGVDMYGLDGEEKWYADFNKQKGVEPL
PPFADPLTYPGGYEQAVANQQICKQNLATSIKANKNPEEKIVPPHSSIYPRDDVVLGVKN
TLICHVSGFHPAPVRVRWTRNNQIVTEGVRMSTPYPNQDFSHNQFSSLTFTPEEGDIYSC
TVEHQGLSEPLTRIWEPEVTQPSVGPAVFCGVGLTLGLLGVATGTFFLIKGNECN
>Eslu-DAA2ps XP_034143102.1 chr.17 NC_047585.1:25.223.821-25.225.006
MFGLDGEEKWYAILSPTLEANLQPCXGNLAVDIKAYKNPEEKIVSPHSSIYPRDDVVLGV
ENTLICHVTGFIPPPVRVRWTRNNQTVTEGVHMSTPYPNQDFSHKQSPXLTFTPEDRDIY
SCTVVHQGLSEPQTRIWKPKVTQPSVDPAVFCGVGLTVGLLGVATGTFFLIKGNERLRLQ
LQSIMVKSV
>Eslu-DAA3 chr.17 XP_034143470.1 NC_047585.1:25.303.871-25.315.116
MICCCLNMKTPMIFFVLASVVYTEGKFLHIDLSIDGCSDSDRVNTIGEDGDELWFADFNK
HQGVGALPPFADPISFQGLYERAVSSQQICKHNLVVDSKAFNKPEEKIVPPHSSIYHRDD
VILGLENTLICHVSGFHPPPVRIRWTRNNQNVTEGIRISNPYPNQDFSYSQFSSLTFTPE
EGDIYSCTVDHKGISEPQTRIWEPEGTHPSVGPTVFCGVGLTLGLLGVATGTFFLIKGNR
>Eslu-DAB2 XP_034143218.1 chr.17 NC_047585.1:25.335.671-25.342.225
MGLININMFLIGILSISHRTDGYFEQLIDQCRFISKDLQDMEFIRSEVFNQVEIIRFNST
VGEFVGYTEYGFSIAKRMNSDTAYLAAIQAERDSVCKPNAWNHYRSIMDKTVKPTLRLRS
VTPPSDRHPAMLICSAYSFYPKPISVSWLRDGHEIKSDVISTEELADRDWYYQIHSHLEY
TPKSGEKISCMVEHISLPEPMVYDWDPSMPESERNKIAIGASGLVLGAIIALAGLVYYKK
KSAGVLP
>Eslu-DBA1 Chr.20 XP_010883054.2 NC_047588.1:30.293.930-30.298.643
MRMNIFVIILILSGTVCTSAQDHELFATLTCFEPSHPELVVMVDGDEVGYTDFQKDEPVM
LLPTPADIHIPFSIVNAYAQLSEMWCKERIAWGELATPSIPQVKDAPESTIYTRNEVELG
VNNTLICFFYNFFPPAIKVNWTKNGMEVTGGASLSRYHPNKDGTFHLFSTLSFTPLEGDI
YACTVEHTALEEPKTRSWEPEVSEFSAGPTIFCGVCLTLGLLGVATGTFLLTKRKPCFKY
FYTQTGRTGISFDLQGVSANL
>Eslu-DBB1 NP_001290706.1 chr.20 NC_047588.1:30.301.515-30.308.996
MCVLYFWSLHLLFFCVVHSSDGVFAEHNARCHFRKVDEIEYTEDIYFNKEVLLWYNGSTG
IWKGDTSSKNILASYFNNDPNDHFSRRAIKDVLCLLNAKALYENLKNYTVKPNVRLKSVE
HSSHRHSAMLVCSAYDFYPKTIMITWLRNGQTVNSDVTSIEEMSNGDRSYQIHSYLEYTP
TAGEIITCMVEHYSLLEPQLHDWDPSMPKSEMNKIVIGISGLLLGLVFLAAGLIYNRKNS
TVLIPVRTSDDMGPSSG
>Eslu-DBA2 XP_034144659.1 Chr.20 NC_047588.1:30.310.087-30.314.003
MNFSVIILILSGIICSFADVEHEVIGAVGCFESGDTKVVVMLDGDEMFFSNYEKEMVETK
MPGSLSGIESPSVQYQYAKLSIRRCKERLEWGRNAVPSIPQVKDAPKSSIYTRNEVELGK
NNTLICFVNGFFPPPINVNWTKNGKDVTEEAALSSYRPNTDGTFHQFSTLSFTPKDGDLY
ACTVKHTALEEHQTRTWETEVSESRGGPAIFCGVGLTLGLLGFSTGTFLLIKGKMKF
>Eslu-DBB2 XP_010883050.2 chr.20 NC_047588.1:30.314.683-30.320.727
MSVLKFLSLHLVLLFSVHAVVFPTDGNFAELHARCRFRKKYDIEYIEENYFNKKKMLFYN
TTKDYWIGYTPSGTDLANIFNKDPYHKDAKRAAKDILCVAHADPLYNFLQNYTLTPHIRL
RSVKPTGTRHSAMLVCSAYDFYPKTIIMTWLKNKRAVTSDVISTEEMSNGDWSYQIHLYL
EYTPTAGEIITCMVEHITLTEPQLYDWDPSMPKSERNEIVIGVFGLLLGVVSVAMGLIYY
KKKSTGLMLVPTGYDMDPSSG
>Eslu-DBA3 XP_019896238.2 chr.20 NC_047588.1:30.322.856-30.325.597
MFHHEIHFIYGCFESGDPEVGMMVDGDEVFYGFFNSNVVDVVWTLPKYPPIKMNQIQKLL
ALEYVTNSEPWCKENLAWGQMAQPKIPKVKEAPESTIYPREHVKLGENNTLICFVNNFFP
PPVKMYWKKNGMEVTKGTSLSRYYPNKDGTFHQFSTLSFTPEEGDIYACTVEHTSLEDPE
SYFWEHEVSEVIVSSAGYSVFCGMGLSLGLLGVATGTFLMVKRSKSN
>Eslu-DBB3 chr.06 XP_010867200.1 NC_047574.1:6.834.048-6.839.218
MSKLINYYIDMLLLFFCLTELVDSSDEDFGYVDAWCRIRSRDLHDLEYILEHHINKQMVA
QYNSTTERWKGYTEYGEMSAKLWNNDPDEIPRRTLEKNVLCRLNAPVIYDNVEAFMDPPN
VTLRQDGPPSNPTLVCSVRYFYPKDITVTWLRNGQEVTSDVTTTGQLANGFWSYQIHSHW
KYTPTAGDRITCMVEHFSLSEPKLYDWDPSLPESERRKIVIGACVLMLGSVFLAAGLVFR
NRSTEWPMETTGAVNYATISG
>Eslu-DCA chr.20 XP_019897056.2 NC_047588.1:30.854.142-30.855.420
MVRNNAQKSRLIRPGNILPVFNCPIFVPHEAIYVFGCREEGDDEAEIQLDGNEIVHADFQ
RREVVWMLPGDTESFSNVYFTHGIFLRDAFRNIQTCRDVLKIWTAEERSPPEVKEAPEST
IYSREDVKLGENNTLICFGNNFFPPPVKMYWKKNGMEVTEGTSLSQYYPNKDGTFHQFST
LRFTPEEGDIYACTVEHTALNKPQTRFWEYKNSEMNGSSAGSAVFCGLGLVLGLLGVASG
IFLYVKGQQFN

>Eslu-DCB XP_034144799.1 chr.20 NC_047588.1:30.855.505-30.860.622
MSGLNLSIIHLLLLFSLSGVVSYFGHIQMKCQFNSKDLRDTELVLNLNINKGLVAEYNSS
TNRFTANTEWVKKLVDSVNDEPDSRHKIQFYFDQYCRRNIPLMYEYIMNKTLKPQVRLSL
AEPSGTRHSAMLVCSAYRFYPKDIIVTWLKNGQKVTSDVSSTEQLADSDWSYQIHSYLEY
TPTAGEIITCMVEHYSLTEPQLHDWDSSIPWSERNNIVIGASVLLLGAVSVAMGLIYYRK
KSTANRTGPI
>Eslu-DDB1 XP_010870891.2 chr.09 NC_047577.1:6.996.793-7.014.272
MMASLQSYLLFVCLFSFFIQTDGFFLTIQHLCYFRGEDPLDVEYVFRIYSNRVNALEYNS
TLNRFSGYTPYGESSASHINQDPLSLTKQKAVLDLYCKHYGPLVYDAVLLKMVQPYVRLS
VEKHPSDRHPTMLKCSAYKFYPRKIRVTWLRDGQEVPMSSTEELANGDWYYQIHSYLEFT
PTPGEKISCMVEHPSLTEPMILHWDESLPESGRSDLVVGVSGLVLGVIFAAAGLIFYHNR
RKTSGDNSM
>Eslu-DDA1 XP_028978174.2 chr.09 NC_047577.1:7.021.282-7.025.210
MCTVFVILHLGVISALVQAQHVFNVMIKRSKTEEFKMTACADGEEYLYVDLDNRKVVITA
PEFGEEIECPICSLAAENGRIYTKELSNIFGLDTPEDKVPPDVMLYPKEEVKQGVNNSLV
CFVNNFFPPPVQVKWTKNDVNITKEVKLSPYFFNTDITFYHFSTLTFDPEDGDLYTCTVE
HPALDEPLTRKWEFLTDHFLPWYKKKNRAFRSKTIFMHDNAPSHAAENTSVSLAAMGIKG
GTLMVWPPSSPDLNPFENLWSILKQTI
>Eslu-DDB2 XP_028978022.2 chr.09 NC_047577.1:7.035.721-7.040.359
MTHLNTENLLVTMLLPMVFSVITIRCLFCHFHSCVHGCTVMMACLRSYLLSIGMISIFTP
NDGHFFTIHHLCFFRGEETEYILREISDMTKVLEYNSTLKRFTGFTRLGIYNAERFNKDP
LLLALLEAVLNFYCKDYRTNISKLDESHVEPSVHLRSMKPQSSTHTTMLKCSAYKFFPKK
IRVTWLRDGQEVPLNMTSTEELANGDWFYQIHSYLLFTPTPGDKISCMVEHPSLTEPMIL
HWDASLPESERNKIMIGVSGLVLGVIFTAAGLIHYHNGRKYSGAIPVTTQ
>Eslu-DDA2 XP_028978025.2 chr.09 NC_047577.1:7.044.026-7.054.127
MSIDGMMSSVFVILLLGVISAQEPAVHVYDVIKACSETEQFNMIILVDDEEYGYVDLKKN
ATVTTLPDFATPVDCPICLTYAEQESQRAEREISIFSKETQEAKVRPDVMLYPKEEVAQG
TRNSLVCFVNNFFPPPVQVKWTKNDENITERAEAGRYATNSDNTFYHFSTLTFTPEEGDI
YTCTVEHPALDNPLTKIWDFEMPPGTSLGPAVFCGLGLTLGILGIATGTFFYVKGNQRQI
>Eslu-DDA3 XP_019905383.2 chr.09 NC_047577.1:7.061.743-7.066.269
MCTVFVILHLGVISALVQAQHVFNVMVLRSETEEFEMTACADGEEYLYVDLDNQKVVITA
PEFSKQIECPVCLNVAEGYIPVGKKHFSDLAFDSPEAKVPPDVMLYPKEEVKQGVNNYLV
CYINNFFPPPVQVKWTKNDVKITKGVKASHYLFNNDITFYHFSTLTFDPEDGDIYTCTVE
HPALDEPVTRKWEFEVPPGPSLDPVVFCGLGLTLGILGIATGMFFCVKGMQLTNVL
>Eslu-DDB3 XP_012990668.2 chr.09 NC_047577.1:7.068.106-7.075.388
MASLQCYLLFVCLLSFFIQTDGHFITIQNLCYFRGEDPLDVEFVYRIYANRVKSFEYNST
LDRFTGYTPNGIDKARILNYDPASLENKRRRLDYYCKDYGQLGYNAVLLKTVKPYARLRT
EKHPSDRHRIMLKCSAYKFYPRKIRVTWFRDGQEVPMNMTSTEELANGDWYYQIHSYLEF
TPTPGEKISCMLKHASLIEPMILHWDEPLPGSGRSKIAMGASGLVLGLVFAAAGLIYYYY
CRLKATGHHYNRP
>Eslu-DDA4 XP_010864729.3 chr.09 NC_047577.1:7.077.058-7.083.387
MSTLLAILYLEVISAYIQAQHVFNVMVLRSDTEGFEMTGDADGEECFYVDVDNQKVVITA
PEFGEQIECPICLNVAERYIPAGKKHFSDLAFDTPEAKVPPDVMLYPKEEVKQGVNNSLV
CFVNNFFPPPVQVKWTKNDVNITKGVKASHYLFNNDITFYHFSTLTFDPEEGDIYTCTVE
HPALDEPVTRKWEFEVPPGPSLDPVVFCGLGLTLGILGLGTGMFFCVKGKQLTNVL

AF1.9 Deduced amino acid sequences from other species used in phylogenies

Spotted gar (*Lepisosteus oculatus;* Leoc-) MHC class II Ensembl sequences (Main text ref. Dijkstra et al.2013):
>Leoc-501A1 MHCIIA JH591501: 64.610-66.685
MSVGWKCLGLLVVMQGAATVAVEHTYEVAYFCQSENRKGSRNEVFDDDEMFHIDPDRKVD
EPRLPEFEKWAWNDSSLIQGALANLGICENHLKGVMKAIPDEPAVKVPPKPAVFPEEPVE
LGWPNTLICALNDFTPPTAQLRWLKNGQPVTSDVSSTDYIPLSSNKFAMFSYLSFTPQEG
DIYTCHVEHTALSEPVSVFWDAEVPTDSDASETAFCAIGLALGILGVVLGTVFLIKAIKF
HS
>Leoc-501B1 MHCIIB JH591501: 70.640-74.060
MRLVLLCSALVLCLTLSGADDFVYFFRSECHFSASDLRDLVYYVRSMTFNGKERVRFNSTV
GKYVGFDTWGEKQANYWNGQKDYIARLSADKDRFCKYNVQLMASGMQDRKIKPEVKIYPA

KTASQGHTHMLVCHAHGFYPRQIAVSWLRNGQPVSSDLTSMSFASDGDWTYQTLLYLEFT
PQGGETFECAVDHVALDGTLKLRWDPAAREAKRNKVIMGASGLVLGLVFAVIGVVYWKRK
TKGHHRLVSSERLVSCVGQVFSGGLVSCPGCALPCAQCLLG
>Leoc-501A2 MHCIIA JH591501: 82.102-84.669
TSHIDINVIGCKSDDPVAQDEAQLDGDEMFYADFDKKEMILTLPAFADSFGVDPGWVQGA
IANRQICINNLEVAIKAENNPPENTDAPVNTIYPRDEVELGKPNTLICLANNFFPPPVKV
RWTKNDVDVSEQATLSRYYPNSDATFYQFSTLSFTPQLGDVYSCSVEHKALPEPKTRIWG
EERSALTSDAN
>Leoc-501B2 MHCIIB JH591501: 95.883-99.847
MLMCSALGFYPKQIKVSWLRDGQTVTSDVTSTEELADGDWYYQIHSHLEYTPRAGESIVC
RVEHSSFATPKELTWDPSMPEADRNKIIIGASGLVLGLVIAAAGGIYYKKKSG
>Leoc-501A3 MHCIIA JH591501: 120.683-125.781
MHGIAVGSIACREISEWKPGRPCQGGRDRAALGWIFRQDGDAPTAMVYPRDSVELGKPNT
LICSVTDFHPMGIGVTWTHNDRPVTEGVTQTTPLSGRDFSFKVFSFLPFTPRLGDVYSCQ
VQHSALPEPLARLW
>Leoc-615A MHCIIA JH591615: 5.333-9.156
MLCAFLTAVLLGASGVLSQAQIRHLDRLLTFCVSNSTEAELEKEHDEDEIFYVDLETKQS
VQRLPEFAEKWHVGPEWPPFAQQEVETCRLYLAAKGEGFPEEKLDPPISRLYSENEVELG
VPNALICFITDFHPAPVKVSWTRNTEPVTQGFNVTQYYSNKDYSLRLFSYLSFTPQAGDV
YSCSVEHRALQEPLTRLWEVEVQSDSEAAETAVCGVGLTLGLLGVAAGTFFLIKGNKCN
>Leoc-615B MHCIIB JH591615: 11.335-16.190
MDSPLHRLAVAVLVLGCTGSRRSVDGNMYQFVHDCEYNDHLEDFLYTRRDIFNKIEILRY
DSNIQTFVGYTPLGIKYAERFNQDKEYLAGLKDDLDNYCKHNAGVYKSTMTDRKVPPSVK
VSATKLLSSKHPTMLVCHVTGFYPQRITVTWLRDGLEIKTDVTSTDLLANGDWTYQVHSH
LELTPRAGETVACRVEHSSLERPLEVTWDPSMPESKKNKIVIGVSGLILGLIITAAGVIY
YKKKSSGRILVPSD

Tetraodon (*Tetraodon nigroviridis;* Teni-) MHC class II Ensembl sequences (Main text ref. Dijkstra et al.2013):

>T19A ENSTNIG00000005593_ENSTNIP00000008293_Un_random:19583822-19591157
MKMLLLILCCILGVSADGQHEDIRIVGCSDFDGEYMHGLDGEELWYADFRRGEGVYAQPD
FIDPIKYEGFYSTAVAVLQICKFNLGIFRKGYKDMPRELDPPTSPMVYTRNEVQLTEPNT
LVCLVTGFYPAPVNVS
>T19B ENSTNIG00000005592_ENSTNIP00000008294_Un_random:19597929-19598797
MASSALRVSLLFLGLSAAGAFEHYGLRRCDFTSAEPKDMEYSLSVYYNKHLMARFSSSVG
KFVGYDKYGQYQADYWNNQSSFLEAMRSSKQRLCQHNIPLWYSHILSKSVLKDVFVYSVA
PPAGGHPAMLVCSVYDFYPKKIKVSWRRDGQEVSHDVTSTDELADGDWFYQLHSHLEYTP
RSGEKISCVVEHASLKTPLVKDWDPSMPEAERNQIAIGASGLILGLILSLAGFIYFQRKS
RGRILVPTN

Fugu (*Takifugu rubripes;* Taru-) MHC class II sequences Ensembl sequences (Main text ref. Dijkstra et al.2013):

>F402A ENSTRUG00000004342_ENSTRUP00000010322_scaffold_402:55188-57563
MMKMLLLILCCVVGASADSQHEDIRILGCSDLDGEFMYGLDSEEVWYADFSKGEGVDINP
PFIDPITYEEGAYSSAVADLQTCKTSLDITRKSLKDMPPERVAPTSPVIYTKEEVQLSQQ
NTLICFVTGFYPAPVNVSWTRNGEHVTQGTSINVPYPNKEGTFTQISRLAFVPQQGDIYS
CRVQHPALSGQDTRMWTVEVQQPGVGPAVFCGLGLTLGLLGVAAGTFFLIKGNECR
>F7533B ENSTRUG00000017449_ENSTRUP00000044727_scaffold_7533:2656-4187
SRMSSSSLRVFLLFISLYTAAGGFQSYVVDSCDFNSTDLKDIEYTRSLYYNRVMYARFSS
RVGKFEGYTKYGLFQADYWNNQSSILEGLRETKESICQPNIKIDYSNILSKSVEPTVRVH
SVVPPAGGHPAMLVCSVYDFYPRYIKVSWQRDGEEVSQDVTSTDELADGDWFYQLHSHLE
YTPRSGEKISCVVEHASLKTPLVKDWGNHLSVCS
>F7533A ENSTRUG00000017444_ENSTRUP00000044708_scaffold_7533:430-1814
MMKMLLLILCCVLGVSADSQHKDIGIVGCSDLDGEFMYGLDSEELWYADFSKGEGVYGTP
PFIDPFRPTAAAVADLQTCKYNLDVDRKGNKDMPTETVAPTSPVIYTKEEVQLSQQNTLI
CFVTGFYPAPVNVSWTRNGEHVTQGTSINVPYPNKEGTFTQISRLAFVPQQGDIYSCRVQ

HPALSGLDTRMWTVEVQQPGVGPAVFCGLGLTLGLLGVAAGTFFLIKGNECR

Medaka (*Oryzias latipes; * Orla-) MHC class II sequences (Main text ref. Dijkstra et al.2013 and Bannai & Nonaka 2013):

>Orla-DAB*21 AGA53814.1 Main text ref. Bannai & Nonaka 2013
MDSSSLCLLFLTMCSADAFLRYDVDRCVFNSTDLKDIEYIYSMYYNKKEFTRFSSSLGKY
VGYTEYGVKTAERANKDTSELSARKAQKETYCKHNIDNWYKNMLSKSVQPRVRVQSLAPS
GGHHPAMLVCSVYDFYPKTIRVSWLRGKEEVSSDVTSTAEMEDGDWYYQIHSHLEYTPRS
GEKISCKVEHASLKDPLVTEWDPSMPESERNKVAIGASGLILGLVLSLAGFIYYKRKARG
RILVPSS
>Orla-DAA*21 AGA53805.1 Main text ref. Bannai & Nonaka 2013
MKMKLLLFVCGILSGTAAVFHEDLAITGCSDSDGEDMYALDGEEVWYADFKKQTGVEPQP
PFVDHVSYPGGYEQAVANQQICRSNLKISRIAMKDLPLERDPPSNVVVYSRDEVELGEQN
TLICHVSGFYPAPVNVSWTKNGERVSGSINIPFPSSDGTFTQISRLPFVPQLGDIYSCSV
EHPALTEVQTKIWDVEKTQPGVGPAVFCGVGLAVGLLGVAAGTFFLIKGNECS
>Orla-DCB*21 AGA53816.1 Main text ref. Bannai & Nonaka 2013
MDSSSLRVFLLILTLYPADGFIHYILNRCLFNSSDLKDIENIYSYYYNKEEFLRFSSSSG
KFVGYTEIGVKTAELANNDPEKMSRRRAEKETFCKPNIDNDYSTILTKSVQPRVRVQSLE
PSGGNHPAMLICSVYDFYPKKIKVSWLQDQEEVSSDVTSTAEMEDGDWFYQIHSYLEYTP
RSGGKISCRVEHISLKDPLITDWDPSMPESEKNKIAIGASGLILGLVLSLAGFVYYKRKT
RGRILVPSS
>Orla-DDA ENSORLP00000016018 Main text Dijkstra et al.2013 Orla-M5A
MSLTSLLLITGAVCASSTTPRHMFHFIYGCYETDEVRVDLVVDDDTIGYADFTKQEMVWC
LPYVPPSGKDLEKEAFKFAKNSIANCHSVLAKAKKADHGTPLRQEPPDLSIYTRYKAEEG
VLDTLFCSANHFYPPTINFTWTKNGAEVTEGLLNLRFSHNKDGTFRGISTLSFTPQRGDV
YSCWVSHEALERPRIITWESRRRRSPARMFFCASLILCLAGIGTGLYFFIKKPNYCCGCQ
>Orla-DDB ENSORLP00000016051 Main text Dijkstra et al.2013 Orla-M5B1
ILSSLIYFYLLFLTSYYSKDQHGYFMFSDFFCYIPSRNPKEVQYLIDWYFNMELTMQYNS
SVGGWTGFTPAGLITAAKFNADKYDVVQRILERELVCQRSVEMVYNGTEEAKVEPNVSLQ
TVEDNDSTLECSALDFYPKHIRLTWFSNGQEVTEGVTFSDVLPNGDWTYQAHTYLTLTPG
KQDHISCMVQHTSLKEPKIYNWEPSMNQTDRDYIIGGVCALLLGAVFLCVGLIHYKQK
>Orla-DEA ENSORLP00000011499 Main text Dijkstra et al.2013 Orla-M16A
SRKQEGFKCSLIHFKTSFFCFVFSVEHEISYFIGCFAEGSTEVLLEFDSEEILYVDFEKE
AVVFTGPSFFKANLSERIQGLTTYKNGKKNRIWCQLADQYFTAETEERDKDPPGEVLIYT
SAEVQPGVENTIICFVNGFYPPSIKVSWTKNGNPVSEGVSHSRYYPNKDQTFHQFSTLSF
TPSWTDVYSCTVEHPALESPKTVLWEPEFHHQHPNLDVFLAASLGLGLLGFAVGTCLIII
ALKRS
>Orla-DEB ENSORLP00000011497 Main text Dijkstra et al.2013 Orla-M16B
TGKSQNCSLGFLPLFLLFSSTNAFYGHGTLKCQFTSSHDLVYLEQVYFNKRLMVQYNSTL
GKYEGYTKKAKDLADGFSKSKPFLEQAVKNREKCRTHMDLVFELQSHPVEPSVRVTPVVR
QGSSHQAMLACSAYNFYPKQIRLTWLRNGEKVINYVTSTEELPDGNWLYQIHSYLEYTPS
PREEITCMVEHASPKEPKLYNWEVLSPPMFGAVRNKIAVGTALLFGSFVFVAGLFFYKRT
T

Stickleback (*Gasterosteus aculeatus;* Gaac-) MHC class II Ensembl sequences (Main text ref. Dijkstra et al.2013):

>Gaac-G131A1 ENSGACG00000000330_ENSGACP00000000421_scf_131:52153-54217
MKTKTMMKMMVVLVLSGVFCVSADGPHKDIAIVGCSDSDGEEMYGLDGEEVWYADFKHGK
GVRPQPSFVDPIDYREGTYETAVGNQQICRNNLKIDLKAFKDFPLEKDPPSSHMIYPKDG
VELGEKNSLICHVTGFYPAPVTFSWTKNQDNVTEGSSRNVPYLNNDGTFNQFSTLEFTPK
LGDIYSCMVEHLALDHPLVKFYDVQVSQPSVGPAVFCGVGLTVGLLGVAAGTFFLIKGNE
CS
>Gaac-G131B1 ENSGACG00000000336_ENSGACP00000000425_scf_131:56853-59742
MAPSFISVSLLFIGLHAADGFMEFVATECVFNSTELKDIEYIQSYYYNKLEYTRFSSSVG
KFVGFTERGVKNAAAWNNNPSYLSRAKAQKEVYCLNHVPVYYNNMLTKSAEPYVRLHSET

PPGGGPLSMLVCSVYDFYPKKIIVRWTRDGRPETTGVTSTDELADGDWYYQTHSHLEYTP
SRSGEKISCVVEHISLSKPLVTDWTDPSMPESERNKVAIGASGLILGLTLSLAGFIYYKR
KARGRILVPSH
>Gaac-GVIIB ENSGACG00000019051_ENSGACP00000025189_groupVII:2444693-2447881
MAPSFHSVSLLFIFLHAAGGFMEFVKDQCVFNSTDLKGIEFIRSTYFNKLQYTKFSSSVG
KFVGFTEQGMKNAAASNNNNMYMASIRAAKETICQPNIQRMYDNILTKSAQPYVRLHSET
PLGGRTSSMLVCSVYDFYPQNIIVRWTRDGRPEATGVSSTDELADGDWYYQTHSYLEYTP
SRSGEKIACVVEHISLSKPLVTDWDPSMPESERNKIAIGGSGLILGLTLSLAGFIYYKRK
GRGRILVPSY
>Gaac-GVIIA ENSGACG00000019052_ENSGACP00000025193_groupVII:2449972-2451242
MKTMKMMMMIVVLVLSGVSAYVPHEAIRISGCSDSDGEEMFGLDGEELWYADFKLGKGVS
LLPSFLDPITYPGGYEVAVAEQQFCRNNLRIDLEAYKFPLERDPPSSHMIFPKDAVELGE
KNSLICHVTGFYPAPVAFSWTKNQENATEGTSRNIPFPNNDGTFNQFSTLEFTPELGDIY
SCMVEHLALDHPLVRFYDVQMSQPSIGPAVFCGVGLTVGLLGVAAGTFFLVKRNECS
>Gaac-GXVIIB ENSGACG00000003680_ENSGACP00000004837_groupXVII:1117113-1119770
MSSSTFLLLLCLSSCSAADGHGYFMYADFWCNMQTARPQQVEYLVDWYFNKEFTMQYNST
VGKWTGFTAAGLVSAAVFNGNHFDVLQRKEERRLICVDNVGHALNATEDNMAAPSVRLAE
ASGSGHNTTLVCSAYDFYPRRIRLAWLRDGQEVTSGATFSEVTTNGNWTYAVHSSLSFTP
GGRDRVSCKVEHAGLQEPALRTWAAHGARDWETGFLVGGVCALLLGAACLSSGLIVHRRK
YSNIS
>Gaac-GXVIIA ENSGACG00000003731_ ENSGACP00000004896_groupXVII:1163942-1165035
MAVTLMLALAAALCTSAAGSSHDFHYTYGCYESGEVRVDVLLDGDVVAYADFGREEVVFL
IPRLPPFLRDLKKLGYEFAKSSFTHCRSVLAKAKRASPNVTIPQDAPVLSVYSRHEGRGG
AANTLFCLADGFYPPSVNFTWTKNGARVTGGVWDLPYGHNRDGTFHRISTLSTTPREGDV
YSCSVEHRAARRPLASSWAEPKEGPSRVSPAAGFFGASLVVCLIGVASGAYFFTKQPNFG

Zebrafish (*Danio rerio;* Dare-) MHC class II Ensembl sequences (Main text ref. Dijkstra et al.2013):

>Dare-D8.35B2 ENSDARG00000053738_ENSDARP00000070311_8:35956359-35959768
MSQKKLYSSLILILALFFVGESANVYYMYRVSRCIFSSSNISAMVYFDRTYFNKNLFIQF
NSNLGRFEGFNEYGLKLAEFWNNGTFVDQEKDVVEFFCKYNSQIYENSILDKAVKPKVKL
SSVTRAGGRQSTVLMCSAYDFYPPHINVFWLRNGEVMTSEVTSTMEMADGDWYYQIHSEL
EYSPKPGERISCVIEHASSNKPMIYDWDPSLPVFERNKISIGVSALVLGIITAAAGIIYY
KKKTTGRTSVVPSRLITLPLMFGR
>Dare-D8.35A2 ENSDARG00000055441_ENSDARP00000072260_8:36283500-36286998
MELYITTLVLTIILSAGAKVVHEDFAIRGCSDTEKEDTYGMDEEELWHADFNQKKGVETL
PDFGDPMTFPGFYEGSEGEMAVCKSNLALRIKGFKSPPPEMDAPQTSIYPKDDVELGVQN
TLVCHVTGFYPPSVSVSWTKNNINVSEDITLSQYRPRIDGTFNIFSTLKFTPAEGDIYSC
MVKHRAIKDQPQTKTWDVDAVLPSVGPAVFCGIGLTLGLLGVAVGTFFLVKGNNCN
>Dare-D8.45B1 ENSDARG00000041705_ENSDARP00000088910_8:45758566-45761489
MNAKINYSLAAVFLSALFETVHAYYTYAQIQCHVSDSLQKIEFIFSVTYNMIELVRYNST
EDTFFGYTAIGQKFAEEYNKDKVLLAQHDFVLNQCRELGDVILPNAVWLAVKPEVIIRSV
TEAKGNRKAVLVCSAYDFYPKGIKLTWMRDDKKVTAELTSSEVMADGHWHYQIHSYLEYF
PQTGEKISCVVDHASSLKPMIYYWDPSLPESERSKIILGAVGLLMGIFTAAAGVIYYKRK
QTGFYRLPVCLLPMETMNDTELQ
>Dare-D8.45A1 ENSDARG00000079593_ENSDARP00000061131_8:45768098-45769605
MFKTFSGITFFIFCLIHTEGQSYPEFGFIETCRGSSYDKIVFILDTEEAAYKDIEKEKLV
FTLPDMANPQYSLNDLRLNDVGIPAFCTEIYHKILSLNVSEDTEPLEPPWTTVYSRNDVK
LNVKNTLICHVTGFFPPPVRVLWTKNNVNVTDGSTISRYYPNKDGTMNVFSRLSFIPEEG
DVYGCSVEHKALQQPQTRTWDVEVQQPSIGPSVFCGVSLALGLFGFATGVLFIAKGKLCK
>Dare-D8.45B2 DCB_ENSDARG00000088872_ENSDARP00000110668_8:45773380-45774524
MQFSRVACLAMILSALLEKVCGNYGYLQSQCRVLSSTKKVELIFSFIFNKIEYIRYNSTD
QKIVGYTEFGEKFVENYKNNTFVLVLAEFGIDNCKKIAKALISDGMLNHVTVKPEVIIRS
VTEAKGNQKAVLVCSAYDFYPKAIKLTWMRNDKKVTADVMSIEEMADGDWYYQIHSHLEY
FPQPGEKISCVVDHASFHKPMIYYWDPSLPETERSKIILGAVGLLMGIFTAAAGVIYYKR
KQTG
>Dare-D8.45A2 ENSDARG00000086294_ENSDARP00000112102_8:45778095-45779781
MLVYSSDHSIVELTQANMLELLCRIMLFIAYWTCSEGQMDLEYGLMESCTGSPDDEIVFT

FNTELAAYLVVVDGTLVLTVPNFIKDYPGFYELPLLDASPATVGLTALCKVVYEKTLNLN
VSEPLEPPWVLLYPRNDVKLNLKNTLICHVTGFFPPPVRVLWTKNNVNVTDGSTISRYYP
DNDRNFNVFSQLSFIPEEGDVYSCSVEHKALQQPQTRTWDVEVKQPSIGPSVFCGVGLAF
GLLSFAAGIFFNAKGKKWM
>Dare-D8.46A ENSDARG00000075932_ENSDARP00000010619_8:47078720-47080882
MEALLRITLICVCVQAKDYHKYGLIGACGDADQEDFIVQFDDEQLAHVDFKEQKDVITLP
EFAGQAVLPPIYVDAKRAEFNCKAYLAVLREVYASSPEVLEPPWSSIYPKSDPQLNLKNT
LICHVTGFFPPPVRVLWTKNNVNVTDGSTISRYYPNKDGTMNVFSRLSFIPEEGDIYSCS
VEHKALQQPQTRTWDVEVKQPSIGPSVFCGVGLALGLLGFATGVFFTAKGNNCN
>Dare-D18B ENSDARG00000056330_ENSDARP00000090055_Chr18:29872428-29875206
MFNVLVIRLALLLPFLLETAHGHYGFVQFTCHMLGSLQNVEVTYSIYFDTTELLRFNSTE
NKAVAYTEYAMKWANDLNKQPKWLHEQVEKNIADCKLFGETYFPLVAKTVKPEVFVRSLR
EASGKRPALLSCSAYNFYPKHIKLTWMRDDKVVTADVMSTKVMADGDWYYQIHSHLEYFP
QPGEKISCVVEHASSHKPMIYYWDSFLTESNKNKIITGAAGIVLGAIMAAGGLIYYKRKH
TVKSHVHVLIY
>Dare-D18A ENSDARG00000093885_ No ENSDARP_18:29867551-29868594
DYHKYGLIGASGDADQEDFIVQFDDEQLAHVDFKEQKDVITLPEFADLAVFQPLYVDAKR
AFNGKAFLAVLREVYASSPEVLEPPLSSIYPKSDPQLNVKNTLICQVTGFFPPPVRVLWT
KNNVNVTDGSTISRYYPNKDGTMNVFS*LSFIPEEGDIHSCLVEHKALQQPQTRTW

Sturgeon (*Acipenser dabryanus/ transmontanus*) MHCII sequences (Main text ref. Chen et al.2020 and GenBank):

>Acda-1 MN249653.1 Acipenser dabryanus MHC class II alpha antigen (Acda-DAA) mRNA, Acda-DAA*1201 allele, complete cds
MKSCVFLAVLLLTVWTAVQAETVSHLYRNLIACQTNNTNTEDERELDQDELYYIDFDKKE
GVLRLPRFSEHWSVGPGQVPRAEENREVCIHNVAGFAKCHEYPPEEQDPPRASVYTENHL
ELGKPNTLICFINGFYPAAIKVSWTKNTLPVTEGVTQTDLYSNKDFTFRMFSYLSFTPEL
GAMYSCQVQHSALPETLTTFWEPDVQTDSDVGETAFCAVGLTLGLLGVAVGTFFLIKGNK
CN*
>Acda-2 MK923687.1 Acipenser dabryanus MHC class II alpha antigen (Acda-DAA) mRNA, Acda-DAA*0601 allele, complete cds
MKSCVFLAALLLTVWTAVQAETVTHLFRALVACQTNGTLPEDDYELDDDELFHIDFDKKE
AVQRIPDFAKYWSPPEGAPARAETDRQTCINNVAVAAKCHKYPPERQVSPRVTLYPEKDL
ELGKPNTLICFITDFHPAAIKVTWTKNTLPVTEGVSLTQYYSNKDFTLQMFSYLSFTPEL
GDVYSCQVQHSALPETLTTFWEPDVQTDSDVGETAYCAVGLSLGLLGVAVGTFFLIKGNK
CN*
>Acda-3 MK923706.1 Acipenser dabryanus MHC class II beta antigen (Acda-DAB) mRNA, Acda-DAB*1001 allele, complete cds
MGTSSLQISSLYTLCLRGLVLVSLMFCCSKTSFGVEGYLMQMMRDCEYSDSSMTDMVLSW
RYVFNQQEVVHYDTKIKKHVGNTACGVKNAEEWNKDKDRMARLLGDVDRYCKYNMGLYRG
GTTEREIPPSVRVSSIKPFSSQHQTMLICYVIGFYPREIKVTWLRNGAKVTADVSSSELL
PDGDWTYQIHSYLELTPQSGDSYTCRVEHSSLAEAIEEKWDPAMPESKRNKIIIGTSGIV
LGLVISAAGLIYYKKKATGRILVPSD*
>Acda-4 MN249661.1 Acipenser dabryanus MHC class II beta antigen (Acda-DAB) mRNA, Acda-DAB*0303 allele, complete cds
MGTSSLQIYSLYTQCLRGLVLVSLLMCSSKTSFGVDGYLYQVMSDCEYSDSSMTDMVYSW
RYVFNQEEYVHYDSKIQKFVGNTACGVKNAEEWNKDKAQLAGLLGEVDRYCKYNAELDLS
FTADRKIPPAVRVRSIKPFSSQHQTMLVCYAFGFYPRDIKVTWLRNGVKVTADVSSSELL
HDGDWTYQIHSYLELTPQSGDSYTCRVEHSSLAEAIEEKWDPAMPESKRNKIIIGASGIV
LGLVIAAAGLIYYKKKATGRILVPSD*
>Actr-DXA White sturgeon, Acipenser transmontanus,DR975335
MSQVAGVCECAVTELARDQTLSLHWKHHIWIQSGLGYCVNMKSCVFLAALLLTVWTAVQA
ETVTHLFRALVACQTNGTLPEDDYELDDDELFHIDFDKKEAVQRIPDFAKYWSPPEGAPA
RAETDRQTCINNVAVAAKCHKYPPERQVSPRVTLYPEKDLELGKPNTLICFITDFHPAAI
KVTWTKNTLPVTEGVSLTQYYSNKDFTLQMFSYLSFTPELGDVYSCQVQHSALPETLTTF
WEPDVQTDSDVGETAYCAVGLXXSPHQR

Fathead minnow (*Pimephales promelas*) MHCII sequence:

>Pipr-DEB Fathead minnow, *Pimephales promelas*,DT151632
MRTFRLTLVLVGFAFCCVSSKDVYYVFQNIVECEYSKVDLSDMVYIIKLVF

```
NQKLLCSYDSRLGKYVGYDEFGIRNADHYNSQGWKMKQRKEELETLCRAN
ARLYVNSTRRKVPPAVTIRPTKKAHYGQLSTLVCHAYNFYPQAINITWLL
DGSEVTGDVISTEFMDNGDWRYQMHSHLDLVLRRGVSVSCRVEHSGLEKP
LVVQWDSTSLDTRIAKLAVGCFSFLLG
```
