# Supplementary material for: Fate of MHCII in salmonids following 4WGD
Source: Immunogenetics. 2020 Nov 23;73(1):79–91. doi: 10.1007/s00251-020-01190-6 (PMC7862078; doi:10.1007/s00251-020-01190-6)
Supplement: Supplementary file 2 — Supplementary file2 (PDF 478 kb) [file 251_2020_1190_MOESM2_ESM.pdf]

Additional file 2 (AF2). Chromosomal orthology

Northern pike (Eslu)	Atlantic salmon (Sasa)	Brown trout (Satr)	Charr (Saal)	Rainbow trout (Onmy)	Coho salmon (Onki)	Chinook salmon (Onts)	Sockeye salmon (Onne)	MHCII genes
6	1b		18b	23	11a	1q	28b	EsluDBB3
	18a		25	1b	4b	6q	10a	
7	13b	(15)	20b	12a	6a	9p	22b	DB
	4b		23	10a	28	30	10b	
9	2b		nw (21)	13a	nw (20b)	32	21a	DD
	12a	(14)	nw (1a)	17b	nw (1b)	2q	nw (15b)	DD
17	12b	(36)	nw (1b)	17a	nw (1a)	nw (2p)	nw (15a)	DA/DD
	22	(nw)	11	7b	5b	7q	2b	DA
18	15a		28b	8a	12a	5p	24b	
	6b		14a	4b	21	18	13a	Onts-DDB
20	5b	(3)	nw (6.1)	2a	nw (13b)	23	14a	DB, DC, DE
	2a	(37)	35	3a	2a	3p	3b	DB, DC, DE

Orthology between Northern pike and selected salmonid chromosomes containing MHCII genes. Data is a summary obtained from main text references Christensen et al.2018b and Sutherland et al.2016. Potential brown trout chromosomal orthology is based on observed data here shown in parenthesis, but lacking published support on orthology. MHCII genes on unplaced scaffolds are shown as “nw” with potential chromosomal orthology shown in parenthesis.
