# Supplementary material for: Fate of MHCII in salmonids following 4WGD
Source: Immunogenetics. 2020 Nov 23;73(1):79–91. doi: 10.1007/s00251-020-01190-6 (PMC7862078; doi:10.1007/s00251-020-01190-6)

Additional file 3 (AF3). DA and DE lineage gene regions

AF3	MHCII regions	Page
AF3.1	DE lineage gene regions	2
AF3.2	DA lineage gene regions	3

Genomic regions with DE and DA lineage genes for Northern pike and selected salmonids are shown as gene boxes within each region. Regional location is shown on the left hand side of each region, while chromosome or scaffold reference is shown below each region. MHCII genes are shown using orange boxes, syntenic genes between pike and salmonids using green boxes, genes with synteny to the mammalian MHC region using cyan boxes and remaining gene boxes are shaded grey. For DE lineage gene regions, homeolog chromosomes are lined vertically for each species while salmonid orthologs are lined horizontally (See Additional file 2). Black lines are used to show regional gaps where gap size is shown in megabases (Mb). Species abbreviations are as follows: Eslu for *Esox Lucius* (Northern pike), Sasa for *Salmo salar* (Atlantic salmon), Onmy for *Oncorhynchus mykiss* (rainbow trout), Onts for *Oncorhynchus tshawytscha* (chinook salmon), Onne for *Oncorhynchus nerka* (sockeye salmon), Onki for *Oncorhynchus kisutch* (coho salmon) and Saal for *Salvelinus alpinus/malma* (charr).

AF3.1. DE lineage gene regions

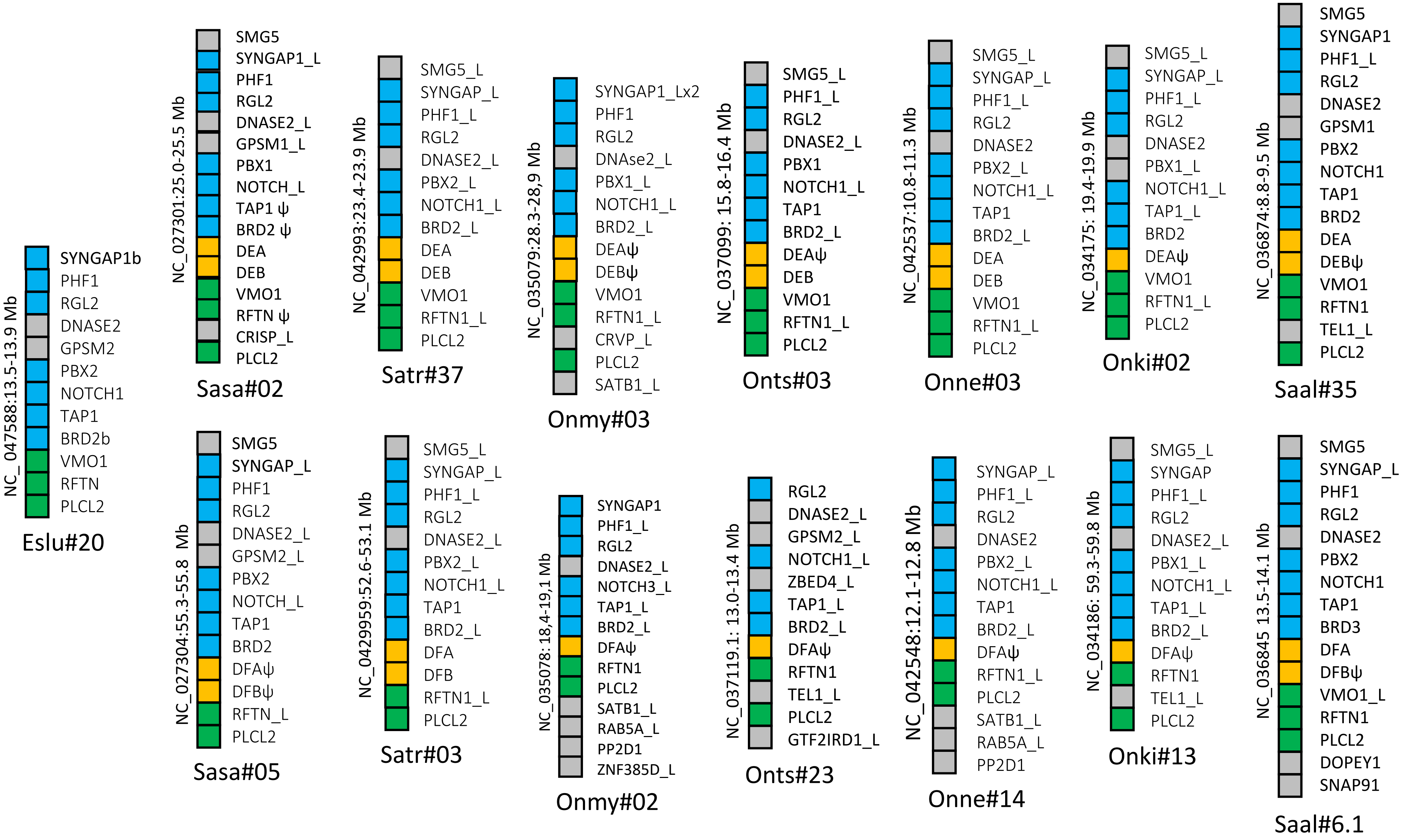

AF3.2. DA lineage gene regions

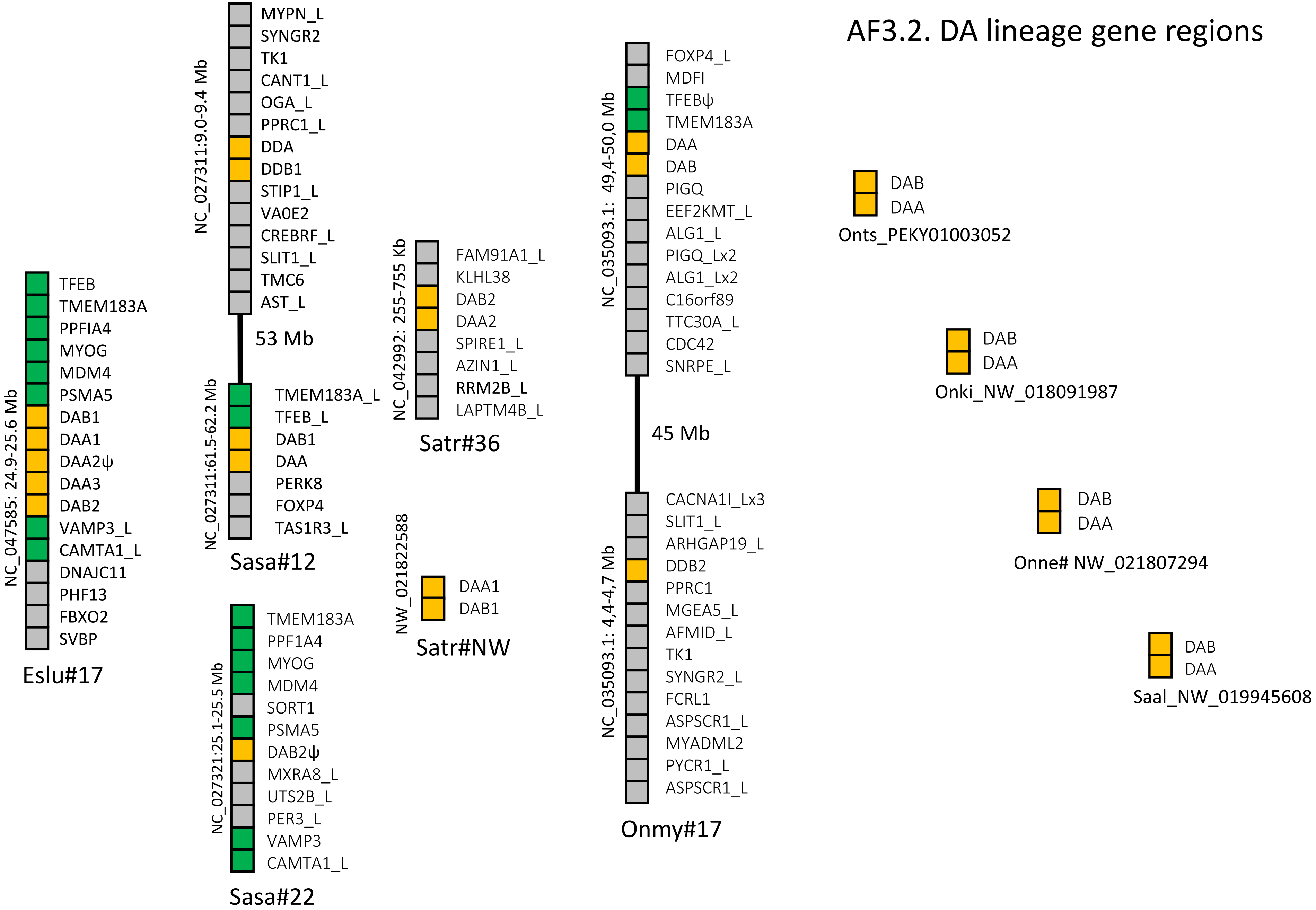

Supplement: Supplementary file 3 — Supplementary file3 (PDF 209 kb) [file 251_2020_1190_MOESM3_ESM.pdf]
