# Supplementary material for: Fate of MHCII in salmonids following 4WGD
Source: Immunogenetics. 2020 Nov 23;73(1):79–91. doi: 10.1007/s00251-020-01190-6 (PMC7862078; doi:10.1007/s00251-020-01190-6)
Supplement: Supplementary file 4 — Supplementary file4 (PDF 666 kb) [file 251_2020_1190_MOESM4_ESM.pdf]

Additional file 4 (AF4). MHCIIA and MHCIIB sequence phylogenies.

Phylogenetic trees of deduced MHCII amino acid sequences from salmonids and Northern pike using Maximum likelihood (ML) and Neighbor-Joining (NJ) methods.

AF	Table of Content	Page
AF4.1	NJ phylogeny of MHCIIA sequences	2
AF4.2	NJ phylogeny of MHCIIB sequences	3
AF4.3	ML phylogeny of MHCIIA sequences	4

Phylogenies were performed using deduced mature extracellular amino acid sequences for both MHC class II alpha (MHCIIA) as well as for MHCII beta (MHCIIB).

Evolutionary analysis by Neighbor-Joining method
The evolutionary history of the phylogenetic trees presented in this supplementary file were inferred using the Neighbor-Joining method [1]. The optimal tree with the sum of branch length is shown. The percentage of replicate trees in which the associated taxa clustered together in the bootstrap test (100 replicates) are shown next to the branches [2]. The tree is drawn to scale, with branch lengths in the same units as those of the evolutionary distances used to infer the phylogenetic tree. The evolutionary distances were computed using the Poisson correction method [3] and are in the units of the number of amino acid substitutions per site. The rate variation among sites was modeled with a gamma distribution (shape parameter = 5). All ambiguous positions were removed for each sequence pair (pairwise deletion option). Evolutionary analyses were conducted in MEGA X [4]

Evolutionary analysis by Maximum Likelihood method
The evolutionary history was inferred by using the Maximum Likelihood method and Whelan And Goldman model [5]. The percentage of trees in which the associated taxa clustered together is shown next to the branches. Initial tree(s) for the heuristic search were obtained automatically by applying Neighbor-Join and BioNJ algorithms to a matrix of pairwise distances estimated using a JTT model, and then selecting the topology with superior log likelihood value. A discrete Gamma distribution was used to model evolutionary rate differences among sites . The tree is drawn to scale, with branch lengths measured in the number of substitutions per site. All positions with less than 95% site coverage were eliminated, i.e., fewer than 5% alignment gaps, missing data, and ambiguous bases were allowed at any position (partial deletion option). Evolutionary analyses were conducted in MEGA X [4]

The following abbreviations are used in phylogenetic trees: Eslu is *Esox Lucius* (Northern pike), Sasa is *Salmo salar* (Atlantic salmon), Onmy is *Oncorhynchus mykiss* (rainbow trout), Onts is *Oncorhynchus tshawytscha* (chinook salmon), Onne is *Oncorhynchus nerka* (sockeye salmon), Onki is *Oncorhynchus kisutch* (coho salmon), Actr is *Acipenser transmontanus (*White sturgeon)*, Acda is Acipenser dabryanus (Dabry's sturgeon),* Dare is *Dario rerio (*Zebrafish), Taru is *Takifugu rubipes* (Pufferfish), Gaac is *Gasterosteus aculeatus* (Three-spined stickleback), Gici is *Ginglymostoma cirratum* (Nurse shark), Lach is *Latimeria chalumnae* (Coelacanth), Leoc is *Lepisosteus oculatus* (Spotted gar), Orla is *Oryzias latipes* (Medaka), Pipr is *Pimephales promelas* (Fathead minnow) and Xipy is *Xiphophorus pygmaeus* (Pigmy swordtail).

1. Saitou N. and Nei M. (**1987**). The neighbor-joining method: A new method for reconstructing phylogenetic trees. *Molecular Biology and Evolution* **4**:406-425.
2. Felsenstein J. (**1985**). Confidence limits on phylogenies: An approach using the bootstrap. *Evolution* **39**:783-791.
3. Zuckerkandl E. and Pauling L. (**1965**). Evolutionary divergence and convergence in proteins. Edited in *Evolving Genes and Proteins* by V. Bryson and H.J. Vogel, pp. 97-166. Academic Press, New York.
4. Kumar S., Stecher G., Li M., Knyaz C., and Tamura K. (**2018**). MEGA X: Molecular Evolutionary Genetics Analysis across computing platforms. *Molecular Biology and Evolution* **35**:1547-1549.
5. Whelan, S. and Goldman, N. (2001). A general empirical model of protein evolution derived from multiple protein families using a maximum-likelihood approach. Molecular Biology and Evolution 18:691-699.

AF4.1 NJ phylogeny of alpha domain sequences

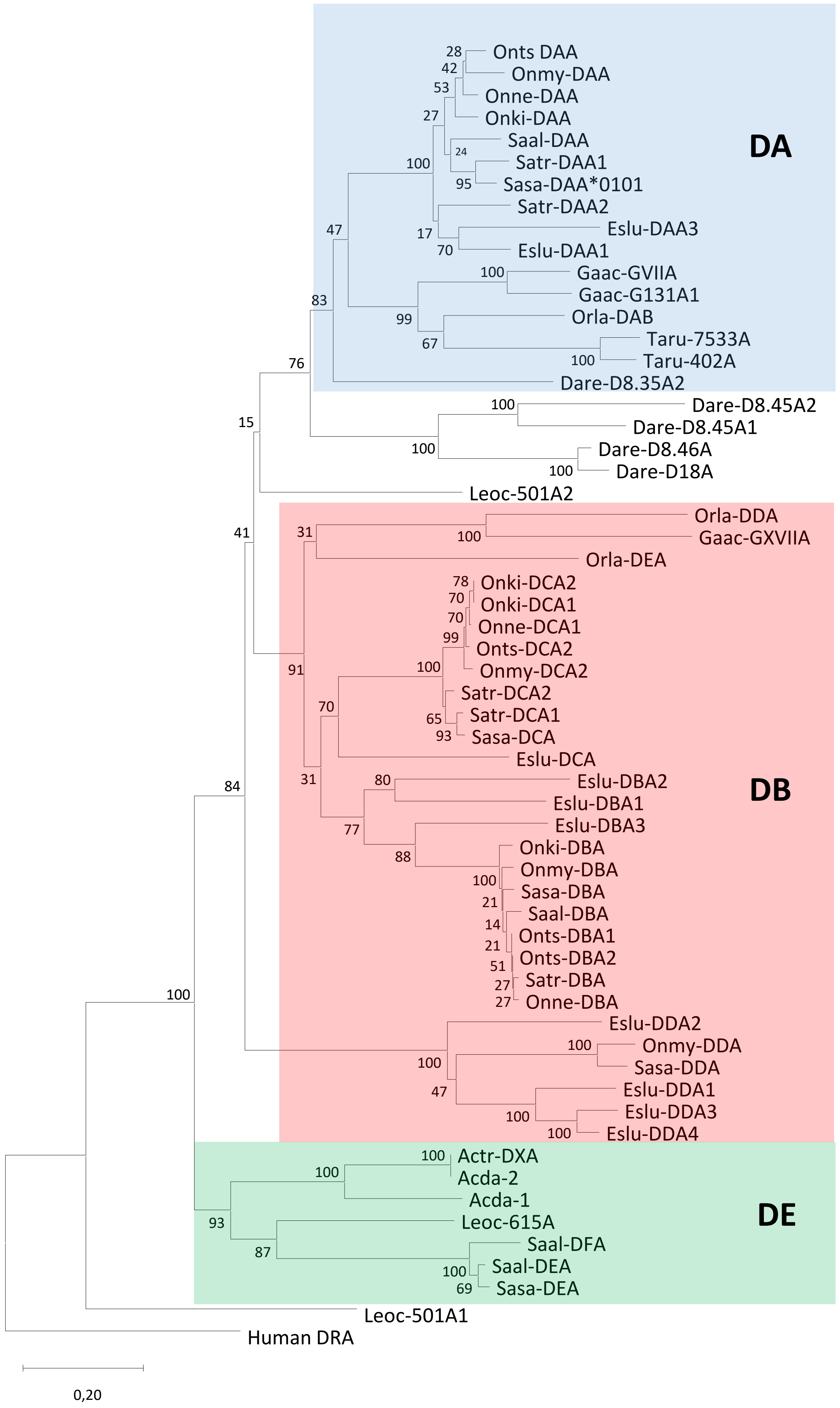

AF4.1 The optimal tree with the sum of branch length = 11,65769766 is shown. This analysis involved 59 amino acid sequences. There were a total of 172 positions in the final dataset.

AF4.2 NJ phylogeny of beta domain sequences

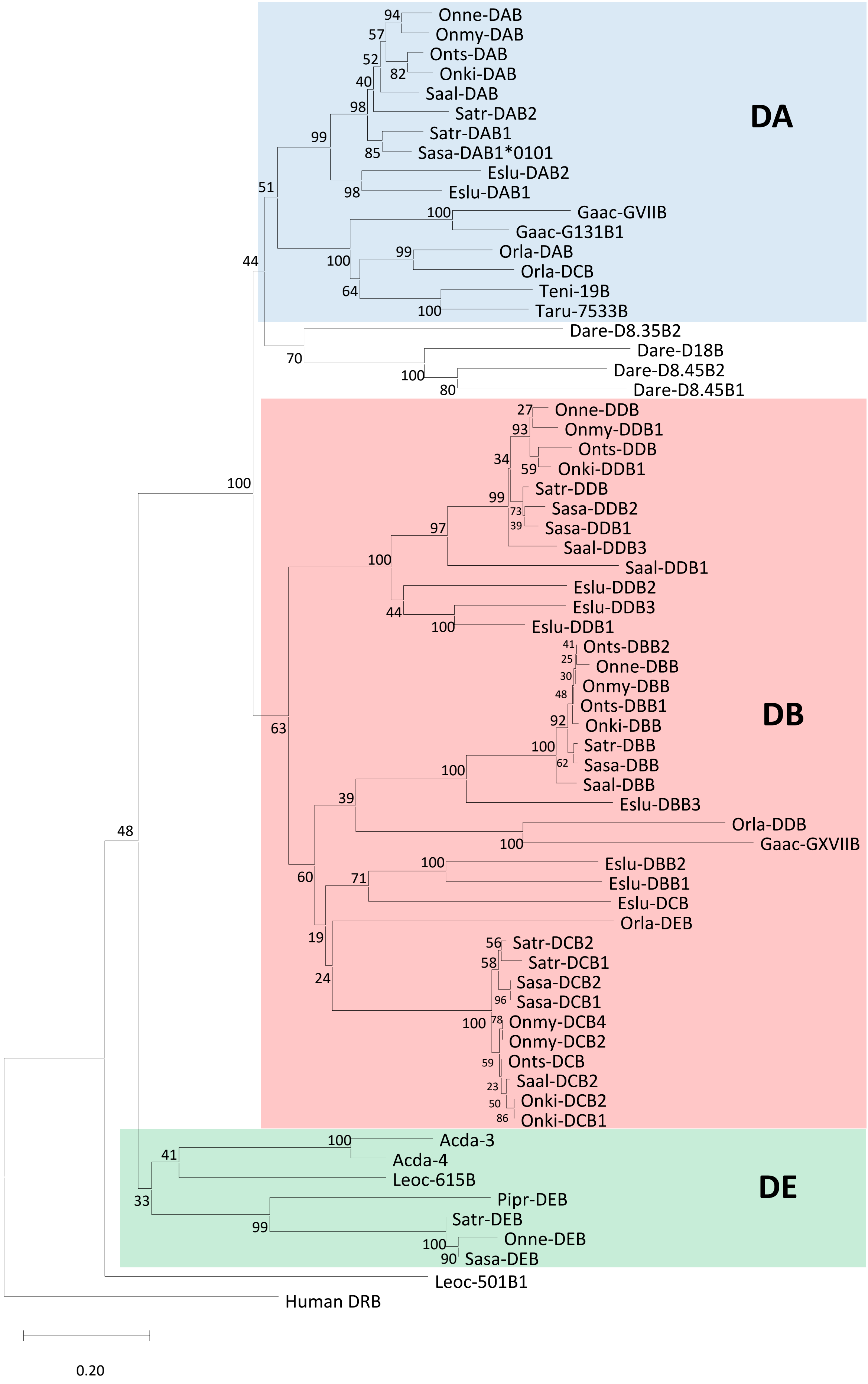

AF4.2 The optimal tree with the sum of branch length = 12.40867953 is shown. This analysis involved 66 amino acid sequences. There were a total of 174 positions in the final dataset.

AF4.3 ML phylogeny of alpha domain sequences

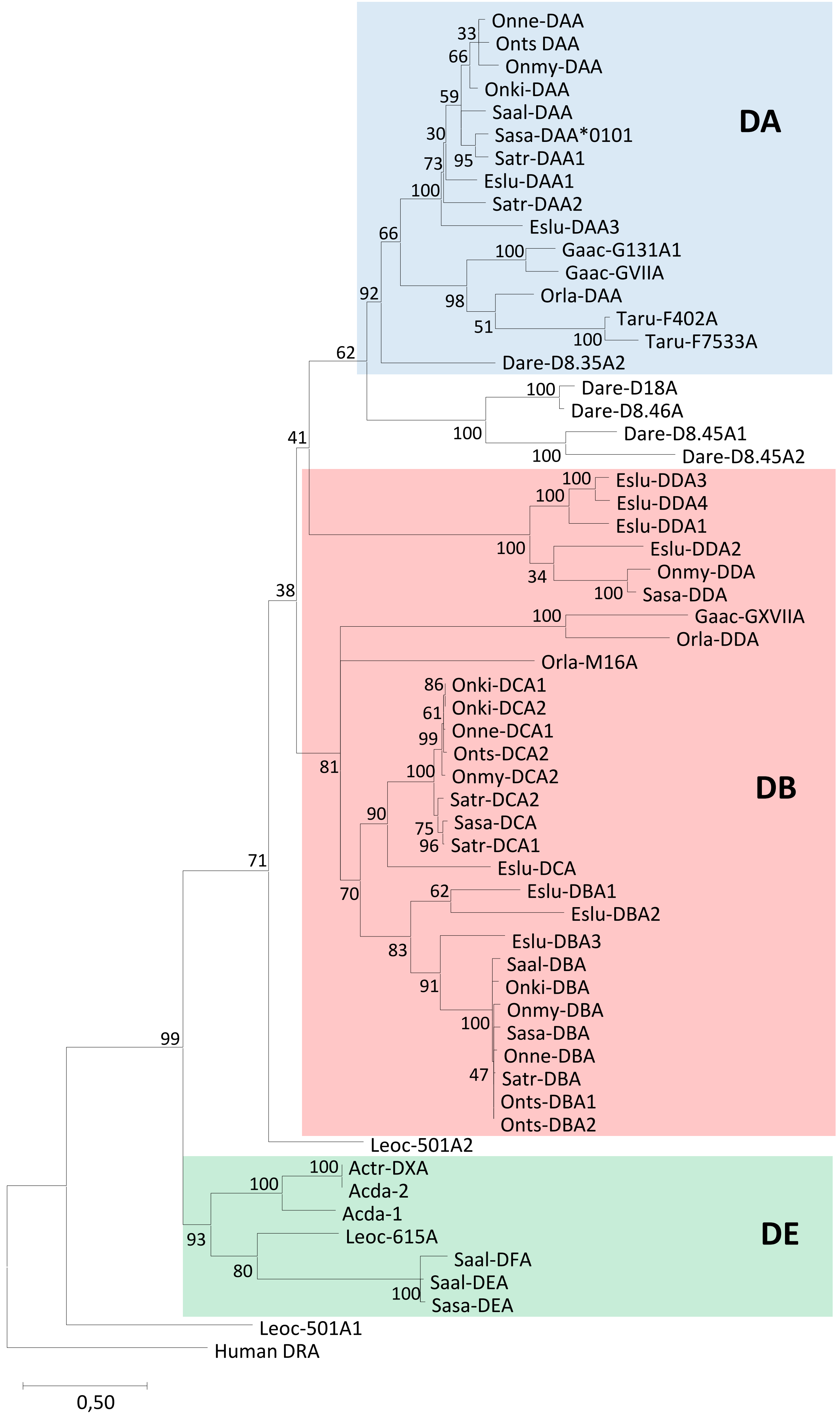

AF4.3. The tree with the highest log likelihood (-9796,78) is shown. A discrete gamma distribution was used to model evolutionary rate differences among sites [5 categories (+G, parameter = 1,3808)]. This analysis involved 59 amino acid sequences. There were a total of 172 positions in the final dataset.
